# Therapeutic potential of cAMP-mediated lysosomal pH modulation in *ATP6V1B2*-related neuropathology

**DOI:** 10.1038/s41420-026-03056-4

**Published:** 2026-03-27

**Authors:** Lu Zheng, Weihao Zhao, Guang Yang, Shiwei Qiu, Yahong Li, Lin Gao, Gege Wei, Ying Ma, Jiangping Xie, Xue Gao, Linyan Chen, Xiaoge Li, Rongfeng Lin, Wei Xiong, Yongyi Yuan, Pu Dai

**Affiliations:** 1 School of Medicine, Nankai University, Tianjin, China; 2 Senior Department of Otolaryngology Head and Neck Surgery, The 6th Medical Center of Chinese PLA General Hospital, Chinese PLA Medical School, Beijing, China; 3 State Key Laboratory of Hearing and Balance Science, Beijing, China; 4 National Clinical Research Center for Otolaryngologic Diseases, Beijing, China; 5 Key Laboratory of Hearing Science, Ministry of Education, Beijing, China; 6 Beijing Key Laboratory of Hearing Impairment Prevention and Treatment, Beijing, China; 7 Department of Pediatrics, The First Medical Center of Chinese PLA General Hospital, Beijing, China; 8 Senior Department of Pediatrics, The Seventh Medical Center of Chinese PLA General Hospital, Beijing, China; 9 School of Life Sciences, Tsinghua University, Beijing, China; 10 Chinese Institute for Brain Research, Beijing, China; 11 Department of Automation, Tsinghua University, Beijing, China

**Keywords:** Hippocampus, Disease genetics, Phenotypic screening

## Abstract

Pathogenic variants in *ATP6V1B2*, which encodes a critical subunit of vacuolar-type H+-ATPases (V-ATPases), disrupt lysosomal acidification via haploinsufficiency and clinically manifest as intellectual disability and seizure disorders. Despite significant morbidity, mechanism-based therapies remain an unmet need. Through integrated clinical analysis of a Chinese cohort and systematic literature review, we delineated genotype-phenotype correlations in *ATP6V1B2*-related syndromes. Isogenic HEK293T models (*ATP6V1B2*^R506X/+^ and *ATP6V1B2*^R506X/R506X^) were generated using CRISPR/Cas9 for dynamic lysosomal pH monitoring via ratiometric RpH-LAMP1-3×flag imaging to evaluate pathophysiological mechanisms. Parallel investigations in *Atp6v1b2*^R506X/R506X^ mice incorporated continuous video-EEG monitoring, behavioral assessments, western blot analyses, and transmission electron microscopy to evaluate therapeutic responses. Drug concentrations in plasma and brain homogenates were quantified by liquid chromatography-tandem mass spectrometry (LC-MS/MS). Clinical analysis revealed central nervous system manifestations (epilepsy, intellectual disability, developmental delay) as primary morbidity determinants. Cellular studies demonstrated significant increase of lysosomal pH in mutant cells compared to wild-type control. Remarkably, treatment with the cAMP analog CPT-cAMP restored lysosomal acidification in a concentration-dependent manner. In vivo studies confirmed spontaneous seizure activity in mutant mice and CPT-cAMP’s penetration of the BBB was confirmed by LC-MS/MS. Intraperitoneal CPT-cAMP administration (20 mg/kg) exerted triple therapeutic effects: (1) significant reduction in seizure frequency, (2) improved cognitive performance in behavioral paradigms, and (3) restoration of autophagic flux through resolution of autophagosome accumulation. These findings establish proof-of-concept for cAMP-mediated lysosomal pH modulation as a viable therapeutic strategy. Our results position CPT-cAMP as a promising candidate for addressing both neurological and cognitive manifestations in *ATP6V1B2*-related disorders.

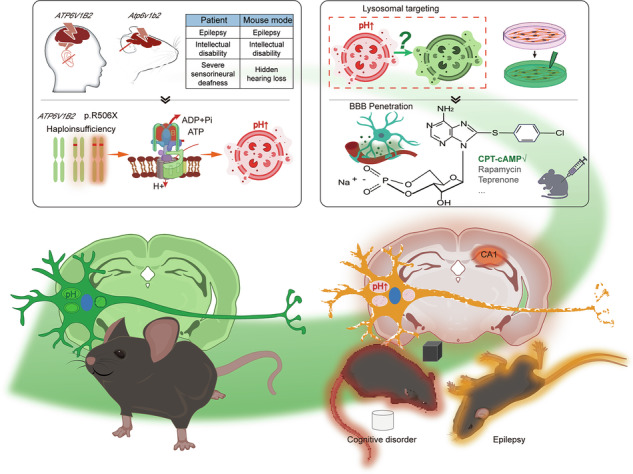

## Introduction

Lysosomes, the principal acidic organelles (pH 4.5–5.0) of eukaryotic cells, orchestrate macromolecular degradation and metabolite recycling through an array of hydrolytic enzymes whose activity is exquisitely pH-dependent [1]. Disruption of lysosomal acid-base homeostasis impairs enzymatic function, leading to pathogenic substrate accumulation and cellular dysfunction [2]. Emerging evidence implicates aberrant lysosomal acidification as a critical pathomechanism in neurodegenerative disorders, including Alzheimer’s and Parkinson’s diseases, where impaired proteostasis and autophagic flux drive disease progression [3].

Central to lysosomal acidification is the vacuolar-type H+-ATPase (V-ATPase), a multisubunit proton pump that utilizes ATP hydrolysis to establish proton gradients across organellar membranes [4]. This molecular machine comprises two structural domains: the membrane-embedded V0 sector facilitating proton translocation, and the cytoplasmic V1 complex responsible for ATP hydrolysis [5]. Genetic perturbations in seven genes encoding V-ATPase subunits have been causally linked to human diseases, with phenotypic manifestations reflecting the spatial expression patterns and functional roles of affected subunits. Notably, homozygous mutations in *ATP6V0A3* cause osteopetrosis with neuroaxonal degeneration [6], while heterozygous *ATP6V1B2* variants underlie multisystem disorders characterized by sensorineural deafness, neurodevelopmental impairment, and connective tissue abnormalities, such as DOORS syndrome (OMIM #220500) [7, 8] and Zimmermann-Laband syndrome type 2 (OMIM #616455) [9], etc.

Our group previously identified the *ATP6V1B2* c.1516C>T (p.Arg506*, p.R506X) nonsense mutation as causative for dominant deafness-onychodystrophy (DDOD) syndrome (OMIM #124480) [10], generating a corresponding knock-in mouse model (*Atp6v1b2*^R506X/R506X^) that recapitulates key neurological features, including seizures and cognitive decline [11]. While inner ear-targeted gene therapy via cochlear perilymphatic route has shown promise for addressing auditory manifestations in murine models [12], neurological complications pose unique therapeutic challenges. Current CNS-directed strategies - whether employing intravenous, intrathecal, or parenchymal delivery routes - are constrained by heterogeneous biodistribution and limited blood-brain barrier (BBB) penetrance. This impasse highlights the need for alternative approaches targeting fundamental disease mechanisms.

As a ubiquitously expressed V-ATPase subunit critical for lysosomal acidification, ATP6V1B2 represents an attractive therapeutic node. Pharmacological modulation of this subunit could potentially restore lysosomal pH homeostasis across both peripheral and CNS compartments. Particularly promising are small-molecule agents with inherent blood-brain barrier permeability, which may circumvent the spatial limitations of gene therapy while minimizing off-target effects. Supporting this concept, lysosomal reacidification strategies have demonstrated therapeutic efficacy in models of metabolic and neurodegenerative disease, restoring autophagic clearance and ameliorating proteotoxic stress [13–15].

The membrane-permeable cAMP analog CPT-cAMP (8-(4-Chlorophenylthio) adenosine 3’,5’-cyclic monophosphate), with the molecular weight of 493.79 Da, emerges as a particularly intriguing candidate. This lipophilic compound activates both protein kinase A and EPAC signaling pathways, with prior studies demonstrating adenosine-mediated V-ATPase trafficking to renal intercalated cell membranes [16]. However, its potential to modulate lysosomal dysfunction and its BBB permeability in genetic models of V-ATPase deficiency remains unexplored.

Through integrated analysis of a Chinese patient cohort and systematic literature review, we elucidate genotype-phenotype correlations in *ATP6V1B2*-related disorders. Furthermore, we demonstrate that CPT-cAMP can permeate the BBB and its administration rescues lysosomal acidification defects, normalizes autophagic flux, and attenuates seizure susceptibility and cognitive impairment in *Atp6v1b2*^R506X/R506X^ mice. Our findings establish pharmacological modulation of lysosomal pH as a viable therapeutic strategy for V-ATPase-related encephalopathies.

## Results

### Clinical findings of patients diagnosed as ATP6V1B2-related syndromes

**Fig. 1:**
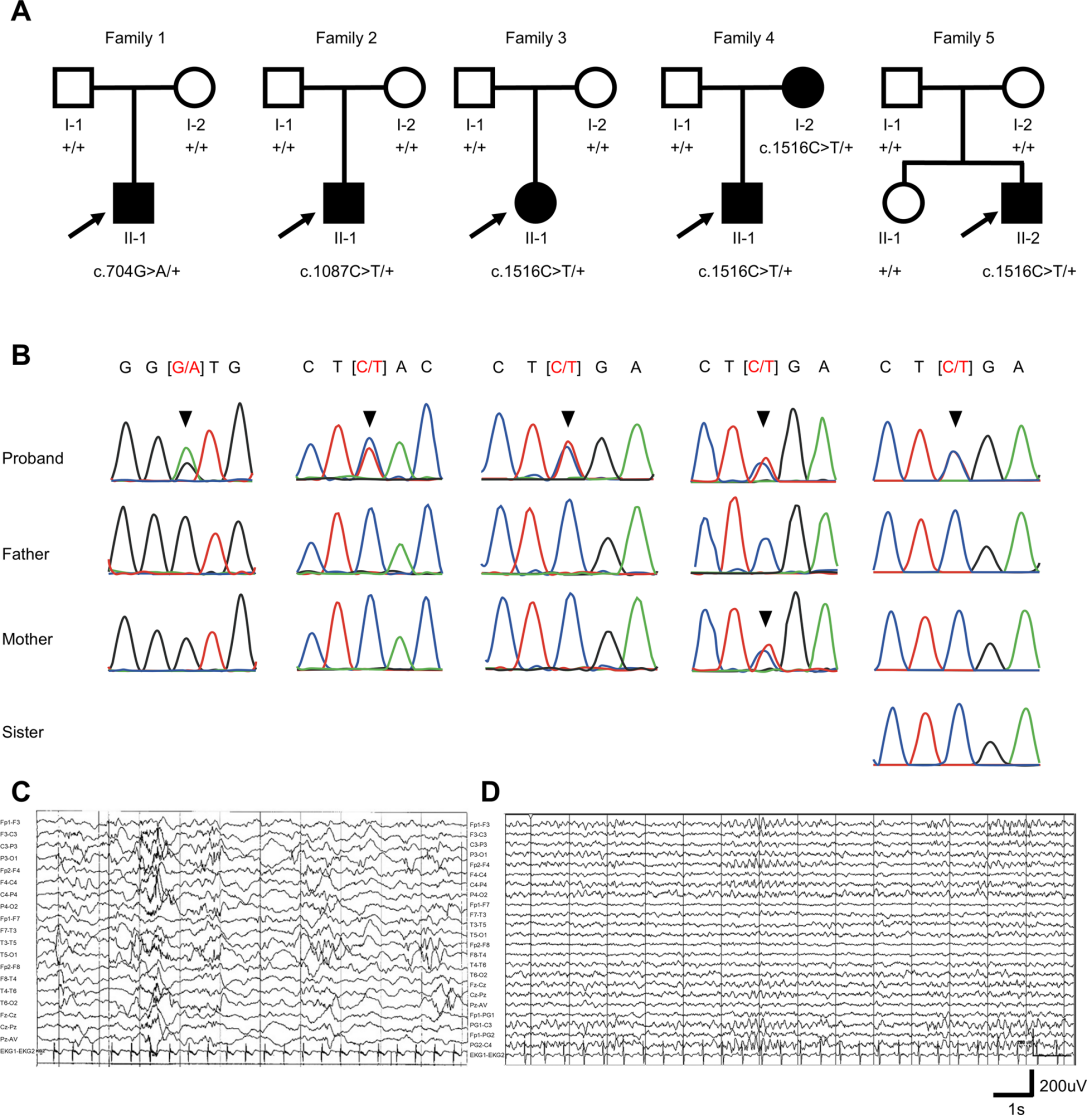
Genetic findings and representative EEGs of cases with *ATP6V1B2* variants. **A** The pedigrees of five unrelated families with *ATP6V1B2* variants. The proband in each family is indicated by an arrow. **B** Sanger sequencing chromatograms confirming the distinct *ATP6V1B2* variants identified in the affected individuals. Five variants were identified, including two missense variants (c.704G>A, c.1087C>T) and one nonsense variant (c.1516C>T). Representative EEGs from Case 1 (**C**), Case 2 (**D**). Interictal EEG of Case 1 (aged 1 year) showing multifocal abnormal discharges, including sharp waves, sharp-and-slow wave complexes, and slow waves with superimposed bursts of fast activity. **D** Interictal EEG of Case 2 (aged 7 months) showing continuous bursts of low-amplitude sharp waves maximal over bilateral frontal, central, and midline regions.

Five Chinese pedigrees including two infantile spasm and three DDOD syndrome harboring *ATP6V1B2* mutations were enrolled (Fig. 1A). Sanger sequencing revealed three distinct variants including two missense variants (c.704G>A[p.Gly235Asp], c.1087C>T[p.His363Tyr]) and one nonsense variant (c.1516C>T[p.Arg506*]) (Fig. 1B). To elucidate the clinical manifestations of *ATP6V1B2*-related syndromes, we systematically summarized genotype–phenotype characteristics of *ATP6V1B2*-related syndromes cases (Table 1). We found that central nervous system phenotypes (epilepsy, intellectual disability, developmental delay) were identified as predominant determinants of morbidity in *ATP6V1B2*-related syndromes. Notably, 69.44% (25/36) of individuals exhibited epilepsy, while 83.33% (30/36) presented with intellectual impairment, underscoring the central nervous system burden associated with *ATP6V1B2* defects.

**Table 1:** Summary of clinical characteristics in reported *ATP6V1B2*-related Cases.

Diagnosis	Variant		Epilepsy	ID	DD	Deafness	
DDOD	*ATP6V1B2* c.1516C>T	p.Arg506*	−	+	−	+	This study
DDOD	*ATP6V1B2* c.1516C>T	p.Arg506*	−	+	−	+	This study
DDOD	*ATP6V1B2* c.1516C>T	p.Arg506*	−	+	−	+	This study
Infantile spasm	*ATP6V1B2* c.1087C>T	p.His363Tyr	+	−	−	−	This study
Infantile epileptic spasm syndrome	*ATP6V1B2* c.704G>A	p.Gly235Asp	+	+	+	+	This study
DDOD	*ATP6V1B2* c.1516C>T	p.Arg506*	−	3/3	−	3/3	Yuan et al. [10]
*ATP6V1B2*-related syndrome	*ATP6V1B2* c.995C>T	p.Ala332Val	+	+	+	NA	Carpentieri et al. [34]
*ATP6V1B2*-related syndrome	*ATP6V1B2* c.982T>C	p.Tyr328His	+	+	+	−	Carpentieri et al. [34]
*ATP6V1B2*-related syndrome	*ATP6V1B2* c.983A>G	p.Tyr328Cys	+	+	+	−	Carpentieri et al. [34]
*ATP6V1B2*-related syndrome	*ATP6V1B2* c.1120G>C	p.Glu374Gln	+	+	+	−	Carpentieri et al. [34]
*ATP6V1B2*-related syndrome	*ATP6V1B2* c.1126C>A	p.Gln376Lys	+	+	+	−	Carpentieri et al. [34]
*ATP6V1B2*-related syndrome	*ATP6V1B2* c.1127A>G	p.Gln376Arg	+	+	+	−	Carpentieri et al. [34]
Lujan–Fryns syndrome?	*ATP6V1B2* c.1121A>G	p.Glu374Gly	+	+	Global	−	Veltra et al. [19]
*ATP6V1B2*-related epileptic encephalopathy	*ATP6V1B2* c.1465A>T	p.Lys489*	+	+	Global	−	Inuzuka et al. [35]
DDOD/DOORS	*ATP6V1B2* c.1516C>T	p.Arg506*	7/9	7/9	7/9	9/9	Beauregard-Lacroix et al. [7]
DOORS	*ATP6V1B2* c.1516C>T	p.Arg506*	+	+	NA	+	Zádori et al. [36]
ZLS-like disease	*ATP6V1B2* c.1192C>G	p.Leu398Val	6/6	4/6	NA	−	Shaw et al. [37]
DDOD	*ATP6V1B2* c.1516C>T	p.Arg506*	−	−	−	+	Menendez et al. [38]
ID with hypotonia and epilepsy	*ATP6V1B2* c.1120G>C	p.Glu374Gln	+	+	+	NA	Popp [39]
ZLS2	*ATP6V1B2* c.1454G>C	p.Arg485Pro	−	2/2	2/2	1/2	Kortüm [9]

*ID* intellectual disability, *DD* developmental delay

### Lysosomal acidification is impaired in *ATP6V1B2*^R506X/+^ and *ATP6V1B2*^R506X/R506X^ cell lines

**Fig. 2:**
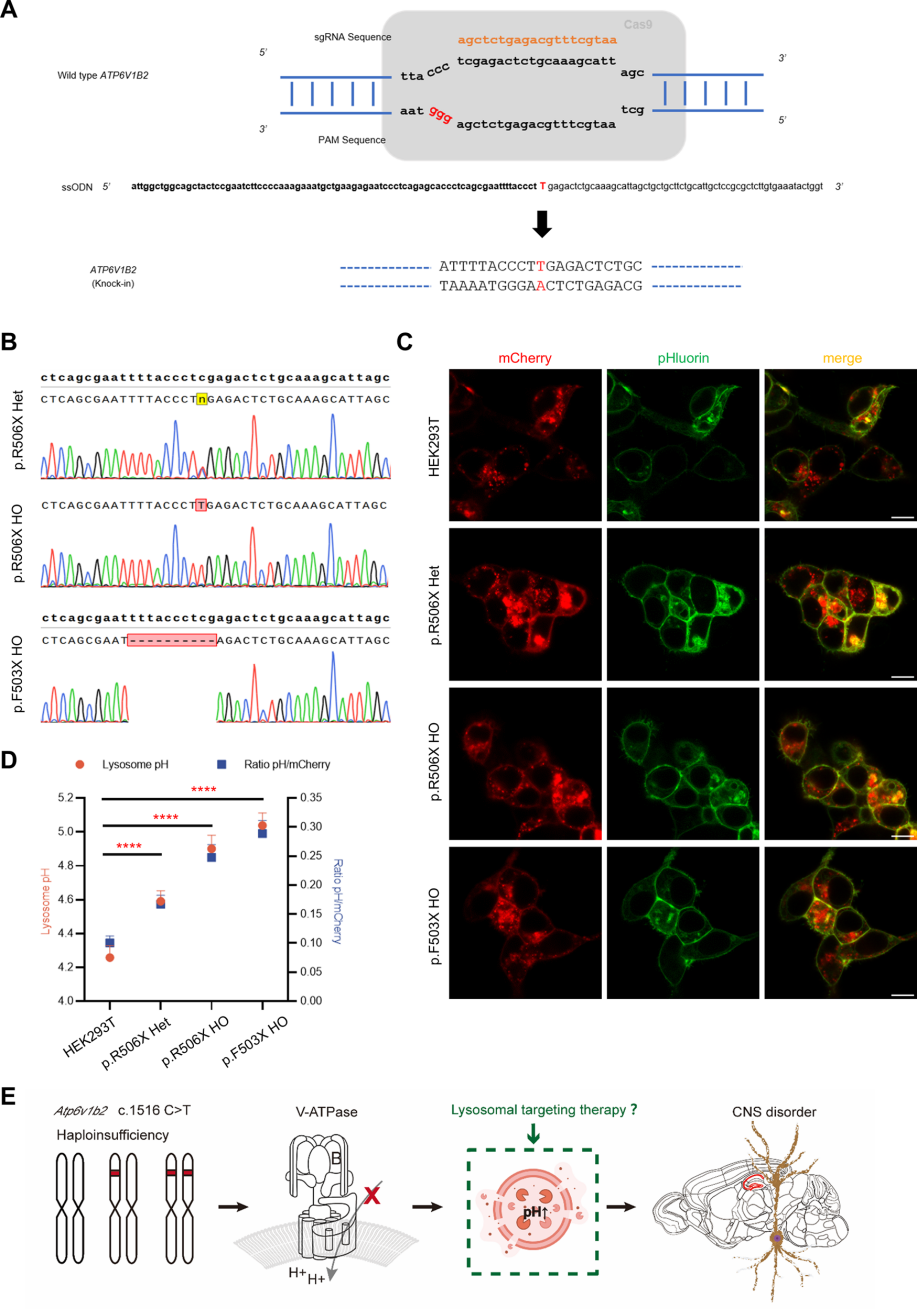
Generation of *ATP6V1B2*^R506X/+^, *ATP6V1B2*^R506X/R506X^, *ATP6V1B2*^F503X/F503X^ cell lines and determination of Lysosomal pH. **A** Design of single-stranded donor oligonucleotides (ssODN) and sgRNAs sequences for targeting the human *ATP6V1B2* gene. **B** Sequencing results of clones show that the c.1516C>T (p.R506X) mutation in *ATP6V1B2* gene and additional 10 bp deletion (p.F503X) was knocked-in in both heterozygous and homozygous state. **C** Expression of RpH-LAMP1-3×FLAG in live imaging in HEK293T, *ATP6V1B2*^R506X/+^, *ATP6V1B2*^R506X/R506X^ and *ATP6V1B2*^F503X/F503X^ showing mCherry and pHluorin channels, merged image. Scale Bar: 10 μm. **D** Ratio and pH calculation for cell lines per mCherry-positive lysosome, pH 4.26 ± 0.07 (HEK293T, *n* = 70), 4.59 ± 0.06 (*ATP6V1B2*^R506X/+^, *n* = 68), 4.90 ± 0.08 (*ATP6V1B2*^R506X/R506X^, *n* = 73), 5.04 ± 0.07 (*ATP6V1B2*^F503X/F503X^, *n* = 66), 6–8 independent fields of view per cell type. (average ± SEM; ^∗^*P* < 0.05, ^∗∗^*P* < 0.01, ^∗∗∗^*P* < 0.001, ^∗∗∗∗^*P* < 0.0001; one way ANOVA). **E** Schematic representation of the pathogenic mechanism by *ATP6V1B2* c.1516C>T variant causing central nervous system disorders.

To investigate the functional consequences of *ATP6V1B2* mutations on lysosomal acidification, we employed CRISPR/Cas9-mediated homology-directed repair (HDR) to generate isogenic HEK293T cell lines carrying specific mutations in the *ATP6V1B2* locus. The editing strategy involved cloning 20-bp sgRNA sequences targeting exon 14 of *ATP6V1B2* into the PX458 plasmid (Fig. 2A). Following co-transfection of HEK293T cells with the pX458-*ATP6V1B2* sgRNA plasmid and single-stranded oligonucleotide (ssODN) donor templates, transfected cells were subjected to single-cell sorting 48 h post transfection to establish clonal populations. Genotypic screening of 56 expanded clones by PCR identified two successfully edited lines. Sanger sequencing confirmed precise introduction of the c.1516C>T mutation in both heterozygous (*ATP6V1B2*^R506X/+^) and homozygous (*ATP6V1B2*^R506X/R506X^) configurations within exon 14 (Fig. 2B). Notably, during clone generation we serendipitously identified an additional mutant line carrying a 10-bp deletion that creates a premature termination codon (designated *ATP6V1B2*^F503X/F503X^), which was included in subsequent analyzes (Fig. 2B).

For quantitative assessment of lysosomal pH, we utilized the ratiometric biosensor RpH-LAMP1-3×FLAG [17]. Cells transiently expressing this pH-sensitive probe were analyzed by calculating the pHluorin/mCherry fluorescence ratio, which was converted to absolute pH values using an established calibration curve (Fig. 2C). The calibration protocol involved treating cells with nigericin and monensin in pH-adjusted calibration buffers (range 4–8), enabling ratiometric measurements across all mCherry-positive organelles (Supplementary Fig. 1A, B). Our measurements revealed significant differences in lysosomal acidification: Wild-type 293T: 4.26 ± 0.07 (*n* = 70), *ATP6V1B2*^R506X/+^: 4.59 ± 0.06 (*n* = 68), *ATP6V1B2*^R506X/R506X^: 4.90 ± 0.08 (*n* = 73), *ATP6V1B2*^F503X/F503X^: 5.04 ± 0.07 (*n* = 66) (Fig. 2D). All measurements were conducted across 6–8 independent fields of view per cell line, with data presented as mean ± SEM. Collectively, our experimental findings demonstrate haploinsufficiency as the pathogenic mechanism underlying the *ATP6V1B2* p.R506X mutation. Given that *Atp6v1b2*^R506X/R506X^ mice recapitulated core neurological phenotypes—including spontaneous seizures and learning-memory deficits, we propose that central nervous system (CNS) disorders similarly stem from lysosomal dysfunction caused by this variant (Fig. 2E). Critically, as lysosomal impairment constitutes a pivotal pathogenic hub upstream in this cascade, screening for pharmacological agents that restore lysosomal pH represents a rational strategy to achieve therapeutic rescue of the underlying CNS phenotypes.

### CPT-cAMP restores normal lysosomal pH in *ATP6V1B2*^R506X/+^ and *ATP6V1B2*^R506X/R506X^ cell lines in a concentration-dependent manner

**Fig. 3:**
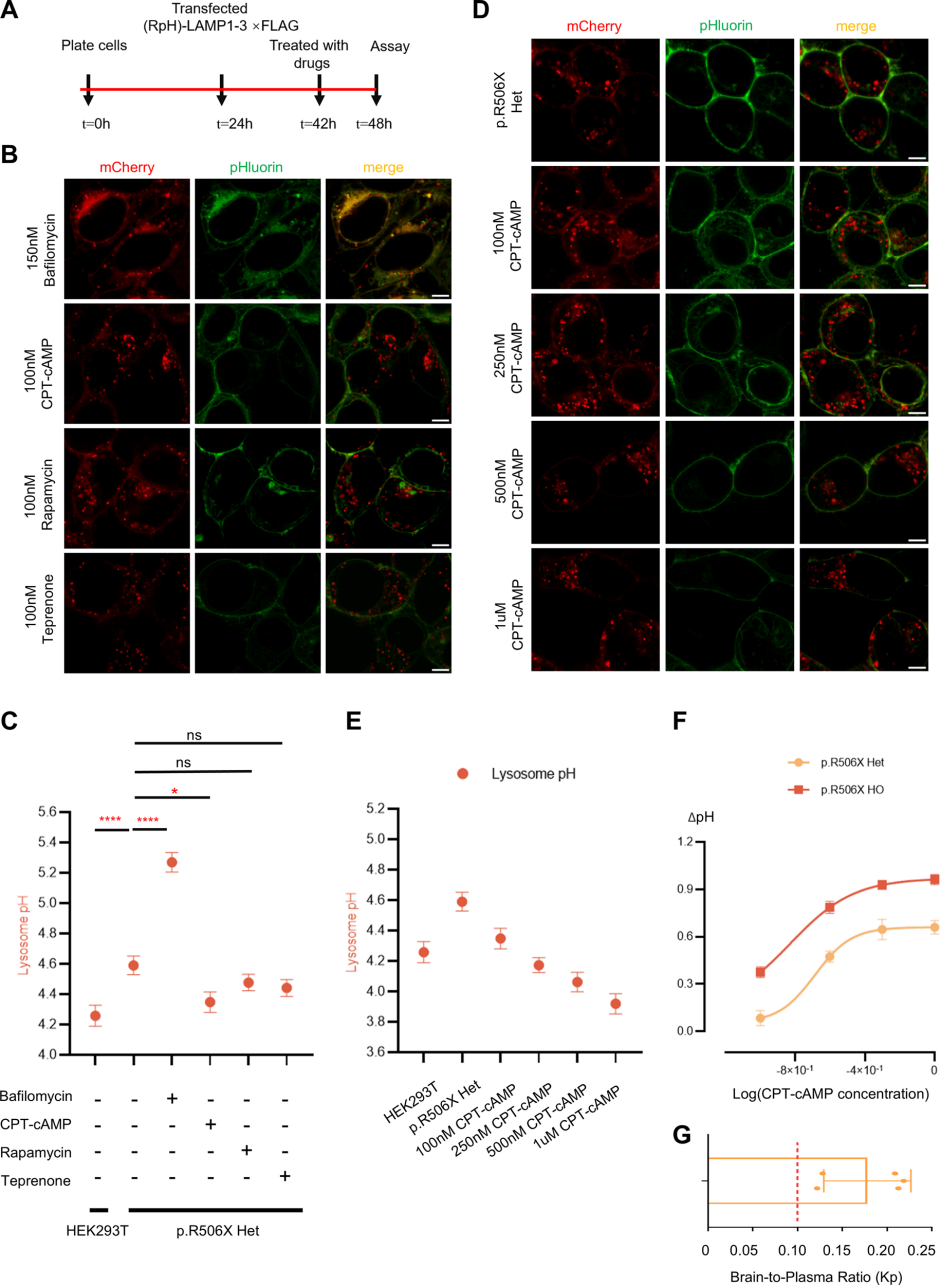
CPT-cAMP rescued abnormal lysosome pH in concentration dependent manners. **A** A schematic of experimental protocol for cell treatment before assaying for lysosomal pH. **B** Expression of RpH-LAMP1-3×FLAG in live imaging in *ATP6V1B2*^R506X/+^ cells dealt with Bafilomycin A1(Baf), CPT-cAMP, Rapamycin and Teprenone. **C** Calculation of pH in *ATP6V1B2*^R506X/+^ cell expression of RpH-LAMP1 -3×FLAG treated dealt with different drugs. **D** Expression of RpH-LAMP1 -3×FLAG in live imaging in *ATP6V1B2*^R506X/+^ cells treated with different CPT-cAMP concentrations showing mCherry and pHluorin channels, merged image. Scale Bar: 5 μm. **E** Quantification of average lysosomal pH in *ATP6V1B2*^R506X/+^ cells treated with different CPT-cAMP concentrations. (*n* = 44–70/group, average ± SEM; ^∗^*P* < 0.05, ^∗∗^*P* < 0.01, ^∗∗∗^*P* < 0.001, ^∗∗∗∗^*P* < 0.0001; one way ANOVA). **F** Dose dependent decrease in lysosome pH in *ATP6V1B2*^R506X/+^ and *ATP6V1B2*^R506X/R506X^ cells treated with different concentrations of CPT-cAMP. **G** Brain penetration of CPT-cAMP. Brain-to-plasma concentration ratio (Kp, mean ± SEM) of CPT-cAMP measured 2 h post-intraperitoneal injection in adult C57BL/6J mice (*n* = 5).

Following the observation of lysosomal pH acidification abnormalities across three engineered cell lines, we systematically evaluated three candidate drugs (CPT-cAMP, Rapamycin, and Teprenone) targeting distinct molecular pathways to identify optimal therapeutic agents for lysosomal dysfunction rescue. Our investigation commenced with live-cell imaging of *ATP6V1B2*^R506X/+^ cells transiently expressing RpH-LAMP1-3×FLAG, using Bafilomycin A1 (Baf) as a positive control (Fig. 3A). Guided by literature-reported drug activities and cellular tolerance data from preliminary assessments [18], we selected 100 nM as the initial screening concentration.

Notably, 100 nM CPT-cAMP demonstrated remarkable efficacy in restoring lysosomal acidification in *ATP6V1B2*^R506X/+^ cells (Fig. 3B, C), achieving significant biological effects without observable cytotoxicity (Supplementary Fig. 2A). To refine dosage parameters, we conducted a concentration gradient analysis (0, 100, 250, 500 nM, 1 μM) revealing dose-dependent pH correction. While 100 nM CPT-cAMP produced statistically significant effects, maximal acidification (0.7 pH unit reduction) occurred at 1 μM (Fig. 3D, E). Intriguingly, *ATP6V1B2*^R506X/R506X^ cells exhibited enhanced sensitivity to CPT-cAMP compared to heterozygous counterparts, demonstrating pronounced pH correction at lower concentrations (Fig. 3F, and Supplementary Fig. 2B). This contrasted sharply with responses to Bafilomycin A1 (a positive control). Here, wild-type cells displayed a more pronounced increase in lysosomal pH than both *ATP6V1B2*^R506X/+^ and *ATP6V1B2*^R506X/R506X^ genotypes (Supplementary Fig. 2C, D), indicating a genotype-specific vulnerability to V-ATPase inhibition. This observed phenotypic divergence can be attributed to a gene dosage effect, wherein homozygous cells exhibit functional disruption of the ATP6V1B2 subunit. This critical deficiency leads to a severely impaired lysosomal acidification capacity, as evidenced by the significant elevation of lysosomal pH (to ~4.90), thereby exacerbating cellular stress and necessitating amplified reliance on cAMP-dependent compensatory pathways to maintain intracellular homeostasis.

**Fig. 4:**
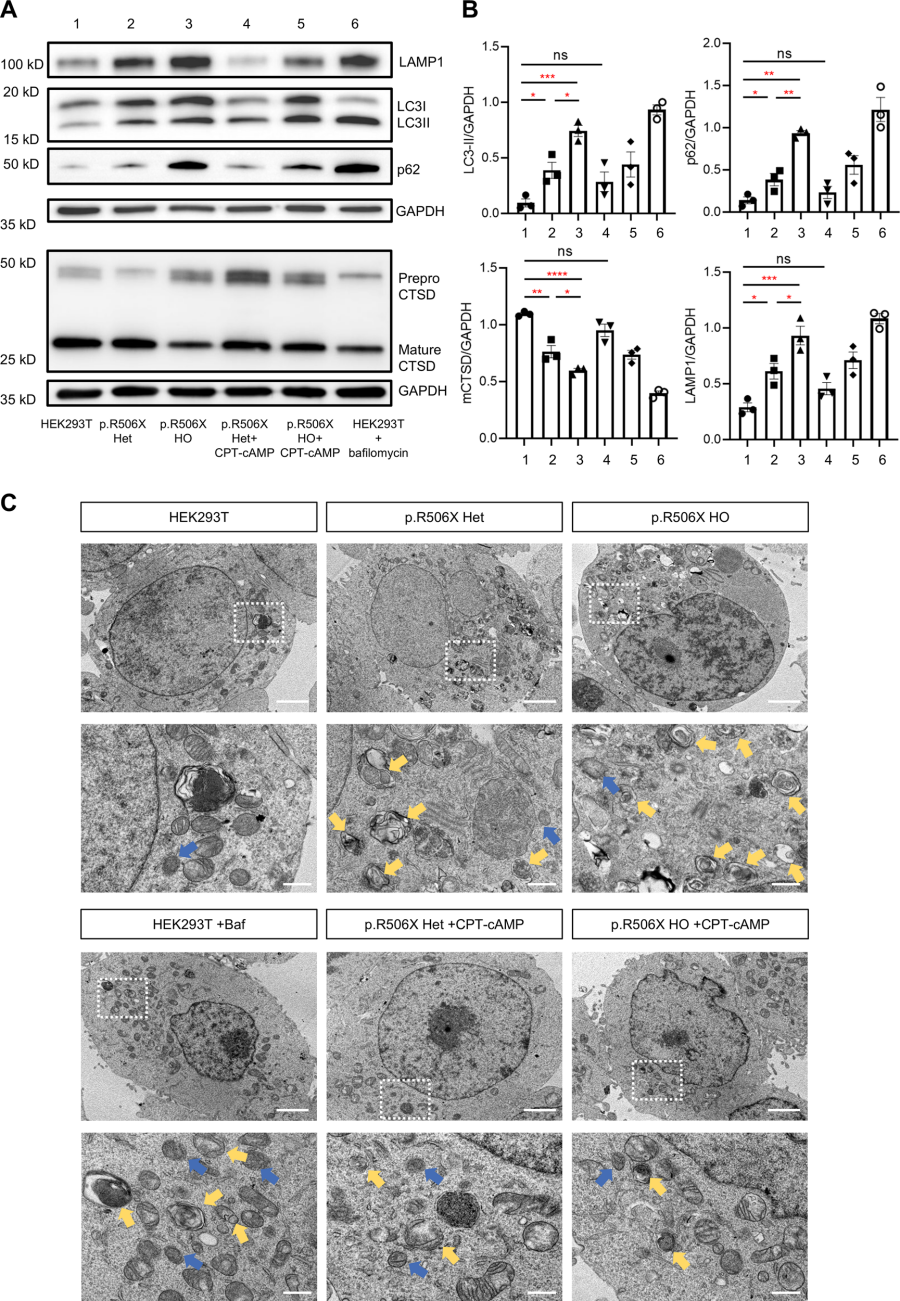
*ATP6V1B2* deficiency causes lysosomal dysfunction and autophagic flux blockade. **A** Representative western blot analysis of autophagy and lysosome proteins in HEK293T (Lane 1), *ATP6V1B2*^R506X/+^ cells (Lane 2), *ATP6V1B2*^R506X/ R506X^ cells (Lane 3), *ATP6V1B2*^R506X/+^ cells treated with CPT-cAMP (Lane 4), *ATP6V1B2*^R506X/R506X^ cells treated with CPT-cAMP (Lane 5) and HEK293T cells treated with Bafilomycin A1 (Baf A1) as a positive control for autophagic flux inhibition (Lane 6). Immunoblotting was performed for LC3I, LC3II, p62, Cathepsin D, and LAMP1. **B** Analysis of LC3, p62, Cathepsin D, and LAMP1 protein expression by quantification protein levels normalized to GAPDH. **C** Representative transmission electron microscopy (TEM) images. Yellow arrowheads indicate autophagosomes and blue arrowheads indicate lysosomes. The increased accumulation of autophagosomes in *ATP6V1B2*^R506X/+^ and *ATP6V1B2*^R506X/R506X^ cells is markedly reduced by CPT-cAMP treatment, providing morphological evidence for the restoration of autophagic flux. Scale bars, 2 μm (top) and 500 nm (bottom). Data obtained from independent experiments (*n* = 3 replicates) are presented as the mean ± SEM. ^*^P < 0.05, ^**^P < 0.01, ^***^P < 0.001, ^****^P < 0.0001 by one-way ANOVA, Tukey’s multiple-comparison test.

To further investigate the lysosomal dysfunction in our mutant cells and evaluate the therapeutic efficacy of CPT-cAMP, we performed a comprehensive assessment using Western blotting and transmission electron microscopy. We analyzed key markers of autophagic flux (LC3-II and p62 accumulation), lysosomal proteolytic capacity (maturation of Cathepsin D), and lysosomal membrane protein (LAMP1 levels). Western blot analysis revealed a significant accumulation of LC3-II and p62 in both *ATP6V1B2*^R506X/+^ and *ATP6V1B2*^R506X/R506X^ cells, accompanied by reduced levels of mature cathepsin D. Concurrently, we observed a marked increase in LAMP1, consistent with lysosomal stress and compensatory adaptation (Fig. 4A, B). This collective profile indicates a profound blockade of autophagic flux and impaired lysosomal function. Crucially, we found that this defect is reversible. Treatment with 100 nM CPT-cAMP successfully reversed these abnormalities, effectively reducing the accumulation of autophagic substrates and promoting the maturation of Cathepsin D and normalized LAMP1 expression (Fig. 4A, B). Consistent with these biochemical findings, transmission electron microscopy (TEM) confirmed the autophagic flux blockade by revealing a substantial accumulation of autophagosomes in both heterozygous and homozygous mutant cells (Fig. 4C). This pathological ultrastructural alteration was markedly alleviated following CPT-cAMP treatment, providing direct visual evidence of restored autophagic clearance. Taken together, these data indicate that CPT-cAMP not only restores pH but also rescues the functional and morphological deficits in lysosomes and autophagy.

Building upon our in vitro findings that CPT-cAMP rescues lysosomal pH abnormalities in *ATP6V1B2*^R506X/+^ and *ATP6V1B2*^R506X/R506X^ cells, we assessed its CNS bioavailability—a critical determinant for treating neurological manifestations. Liquid chromatography-tandem mass spectrometry (LC-MS/MS) confirmed CPT-cAMP’s penetration of the BBB, demonstrating a brain-to-plasma concentration ratio Kp of 0.18 ± 0.04 at 2 h post intraperitoneal administration (20 mg/kg) (Fig. 3G). Collectively, these findings demonstrate strong translational potential of CPT-cAMP for CNS disorders.

### *Atp6v1b2*^R506X/R506X^ mice displayed spontaneous seizures and heightened seizure susceptibility to PTZ

**Fig. 5:**
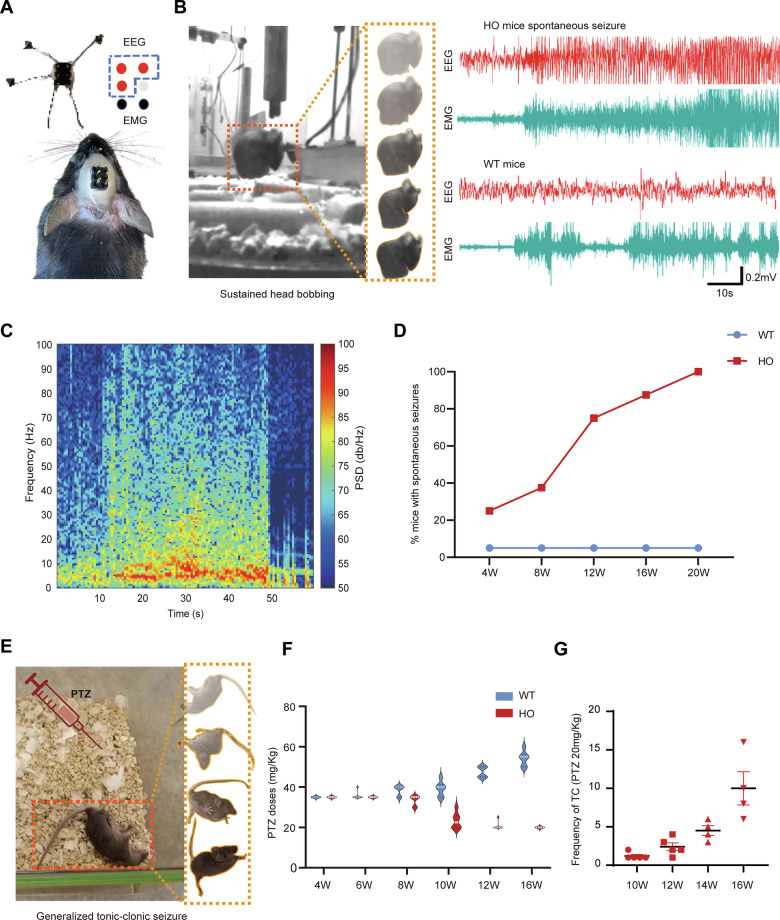
Spontaneous seizures and heightened seizure susceptibility in *Atp6v1b2*^R506X/R506X^ mice. **A** Schematic diagram depicting the electrode schematic and photograph showing a representative mouse with chronically implanted electrodes secured to the skull with dental acrylic cement. Three skull screw electrodes (red) with connected insulated lead wires targeted the cortex for electroencephalography (EEG), two wire electrodes (black) were inserted into the muscle for electromyography (EMG). Electrode connectors are visible on the headstage. **B** Spontaneous epileptic seizure with sustained head bobbing in *Atp6v1b2*^R506X/R506X^ mice (left) and synchronized EEG/EMG recording during seizure onset (right top). Baseline recording from a WT littermate (right bottom). **C** Time-frequency analysis of ictal discharges in *Atp6v1b2*^R506X/R506X^ mice during spontaneous seizures. **D** Percentage of mice displaying spontaneous seizures up to 20 weeks in WT (*n* = 4) and *Atp6v1b2*^R506X/R506X^ mice (*n* = 8). **E** Representative PTZ-induced *Atp6v1b2*^R506X/R506X^ mice exhibiting generalized convulsions with tonic extension and loss of posture. **F** PTZ doses required to induce systemic tetanic convulsion in *Atp6v1b2*^R506X/R506X^ and WT mice (*n* = 6) at different ages. **G** Frequency of systemic tetanic convulsion induced by 20 mg/kg PTZ in *Atp6v1b2*^R506X/R506X^ mice from 10 weeks of age onward.

While patients carrying the *ATP6V1B2* c.1516C>T mutation have not yet exhibited epileptic manifestations, our experimental findings suggest potential epileptogenic consequences of *ATP6V1B2* dysfunction. Notably, other pathogenic variants in *ATP6V1B2* have been clinically associated with epilepsy [19], and our *Atp6v1b2*^R506X/R506X^ murine model demonstrated spontaneous seizure activity, suggesting that impaired ATP6V1B2 function may represent an underrecognized epileptogenic mechanism. Comprehensive behavioral characterization through continuous video monitoring revealed that homozygous mutants developed spontaneous seizure phenotypes ranging from myoclonic jerks to generalized tonic-clonic seizures (GTCS). To quantitatively assess ictal burden, we conducted synchronized video-electroencephalography (EEG) monitoring in 4–20 week-old mutants using a continuous video-EEG monitoring system. Over 72-h recordings, *Atp6v1b2*^R506X/R506X^ mice displayed 1-2 stereotyped electrographic seizures characterized by abrupt tonic spike discharges with progressive frequency and amplitude modulation (Fig. 5B). Spectral analysis of ictal events demonstrated significant energy redistribution within the 1–10 Hz frequency band (Fig. 5C). The incidence of spontaneous seizures exhibited an age-dependent escalation, reaching 100% penetrance by 20 weeks of age (Fig. 5D).

To establish a standardized platform for therapeutic evaluation, we compared pentylenetetrazole (PTZ) seizure susceptibility across genotypes. Video-EEG quantification revealed pronounced genotype-dependent differences in convulsive thresholds. Wild-type mice showed developmental resistance to PTZ-induced GTCS, requiring progressively higher doses from 35 mg/kg (4 weeks) to 55 mg/kg (16 weeks). Conversely, homozygous mutants exhibited paradoxical hypersensitivity with decreasing thresholds from 35 mg/kg (4 weeks) to 20 mg/kg (16 weeks) (Fig. 5E, F). Notably, administration of 20 mg/kg PTZ to mutants from 10 weeks onward induced progressively severe GTCS clusters culminating in lethal status epilepticus (Fig. 5G). These findings collectively demonstrate enhanced limbic hyperexcitability and reduced seizure thresholds in *Atp6v1b2*^R506X/R506X^ mice compared to wild-type controls.

### Therapeutic effects of CPT-cAMP in *Atp6v1b2*^R506X/R506X^ mice

**Fig. 6:**
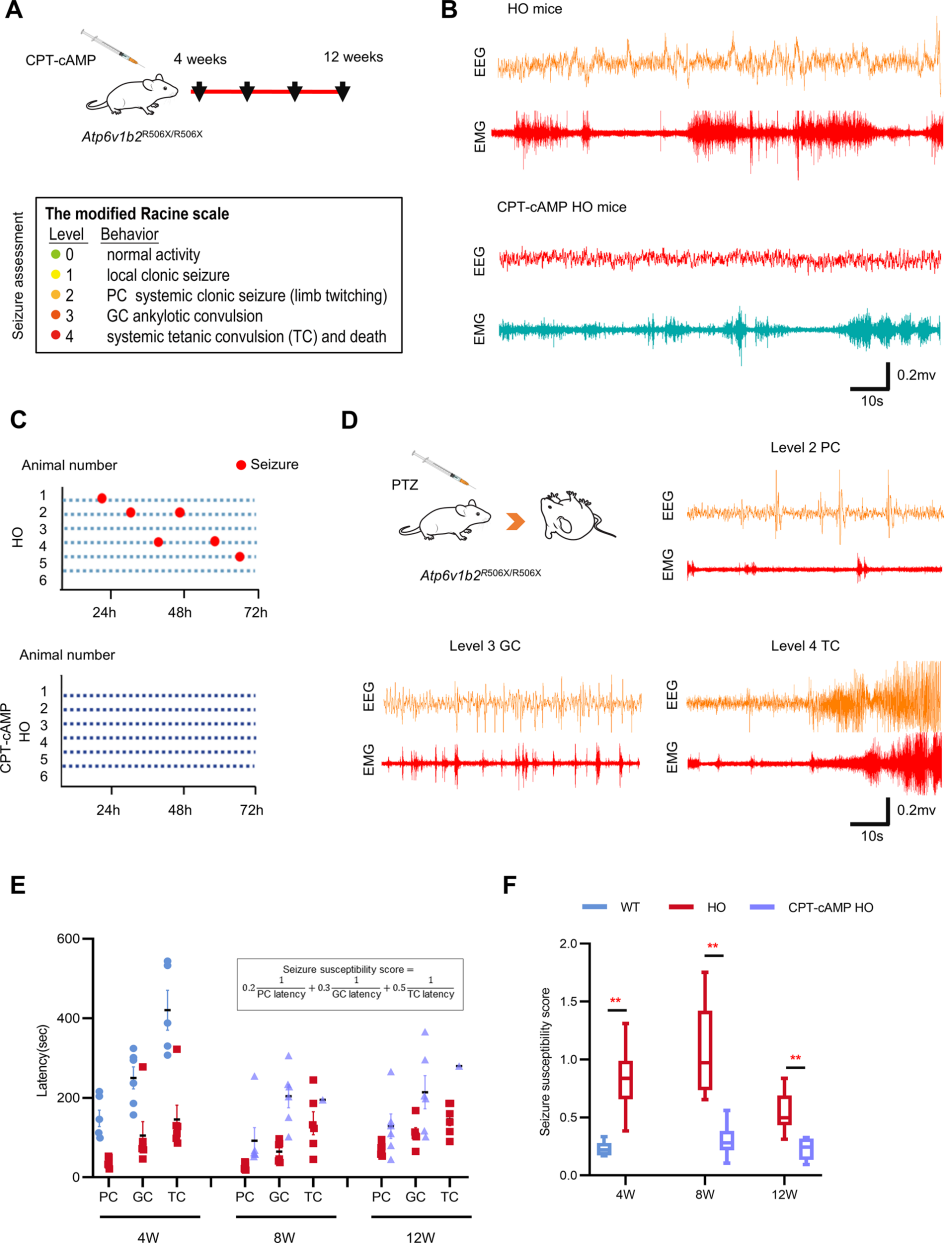
Treatment with CPT-cAMP attenuated seizure susceptibility in *Atp6v1b2*^R506X/R506X^ mice. **A** Schematic of in vivo pharmacological intervention. *Atp6v1b2*^R506X/R506X^ received weekly intraperitoneal (i.p.) injections of CPT-cAMP (20 mg/kg) (Top). Seizure severity classification following Pentylenetetrazol (PTZ, i.p.) induction using the modified Racine scale (Bottom). **B** Representative EEG and EMG trace recordings from *Atp6v1b2*^R506X/R506X^ mice and CPT-cAMP treated *Atp6v1b2*^R506X/R506X^ mice. **C** Continuous 72-h EEG recordings in *Atp6v1b2*^R506X/R506X^ mice. Untreated homozygous mice (*n* = 6) showing representative electrographic seizure events, 66.7% (4/6) exhibited spontaneous seizures (mean frequency: 1–2 events/72 h) (Top). No electrographic seizures detected in any CPT-cAMP treated *Atp6v1b2*^R506X/R506X^ mice (*n* = 6, Bottom). **D** Schematic representation of PTZ-induced seizure model and representative EEG and EMG trace recordings at part clonic (PC), general clonic (GC) and tetanic convulsion (TC) of modified Racine’s scale. The latencies of part clonic (PC), general clonic (GC) and tetanic convulsion (TC) of modified Racine’s scale (**E**), and seizure susceptibility score (**F**) between CPT-cAMP treated *Atp6v1b2*^R506X/R506X^ mice and *Atp6v1b2*^R506X/R506X^ mice.

Following the demonstration of CPT-cAMP’s therapeutic potential in *ATP6V1B2*^R506X/+^ cell lines, we extended our investigation to evaluate its efficacy in vivo using the established *Atp6v1b2*^R506X/R506X^ mouse model. Prior to therapeutic assessment, we established safety parameters through weekly intraperitoneal administration of 20 mg/kg CPT-cAMP from 4 to 12 weeks of age. This regimen showed excellent tolerability, with no significant body weight alterations observed in treated animals compared to controls (Fig. 6A Top, and Supplementary Fig. 3A). To assess therapeutic outcomes, we performed 72-h continuous EEG monitoring on 12-week treated cohorts. Strikingly, CPT-cAMP administration completely abolished spontaneous epileptic seizures during the monitoring period, contrasting with seizure events recorded in untreated littermates (Fig. 6B, C). Given the variable spontaneous seizure incidence in untreated controls - potentially attributable to environmental influences, biological variability, or temporal monitoring constraints - we employed a standardized PTZ challenge (20 mg/kg) to objectively evaluate seizure susceptibility.

Quantitative analysis using a modified Racine scale revealed CPT-cAMP’s significant anti-epileptogenic effects. Treated animals exhibited: (1) Reduced seizure severity scores, (2) Prolonged latency to myoclonic jerk onset (MJ), and (3) Delayed progression to generalized tonic-clonic seizures (GTCS) compared to untreated controls (Fig. 6A Bottom, 6D, E, F). These findings collectively demonstrate that CPT-cAMP administration effectively attenuates both spontaneous and chemically-induced epileptiform activity in *Atp6v1b2*^R506X/R506X^ mice. Our results align with the mechanistic hypothesis that restoration of lysosomal pH homeostasis through CPT-cAMP treatment underlies the observed phenotypic amelioration.

**Fig. 7:**
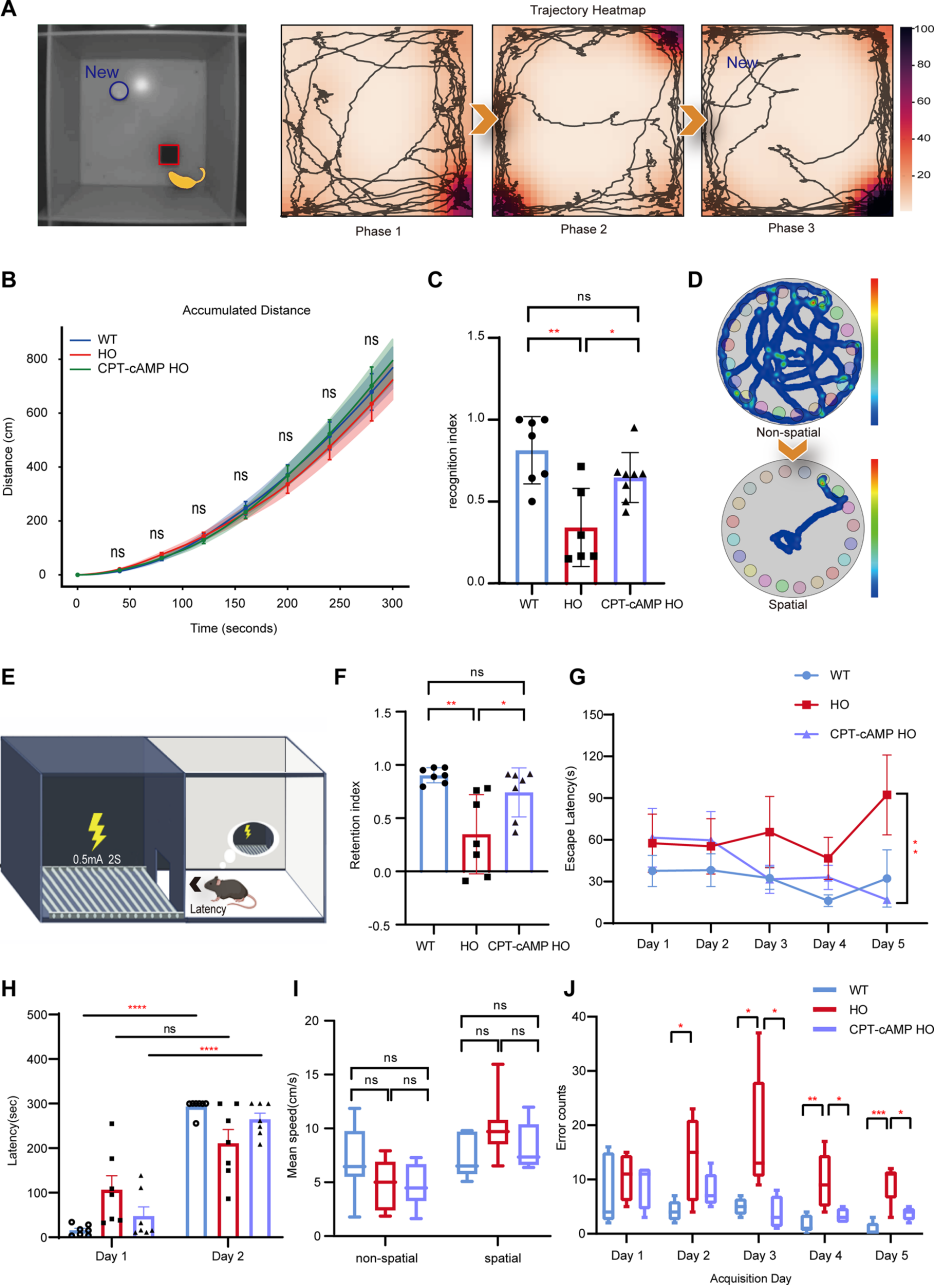
CPT-cAMP treatment attenuated learning and cognitive impairments in *Atp6v1b2*^R506X/R506X^ mice. **A** Mouse annotated with yellow segmentation masks via SAM-based tracking throughout the novel object recognition (NOR) paradigm (left) and representative trajectory heatmaps across three phases: habituation, training, and test (right). **B** Total distances traveled over time in phase 1 in wild-type (WT), untreated *Atp6v1b2*^R506X/R506X^, and CPT-cAMP treated *Atp6v1b2*^R506X/R506X^ mice. No significant differences were observed among three groups, indicating that CPT-cAMP does not alter locomotor activity (*n* = 6-8 mice/group, *P* > 0.05, one-way ANOVA). Data are presented as mean ± SEM, n.s., not significant. **C** Novel object recognition index across experimental groups. *Atp6v1b2*^R506X/R506X^ mice exhibited a significantly reduced recognition index compared to WT mice (^∗∗^*P* < 0.01), indicating impaired learning and memory. CPT-cAMP treatment improved the recognition index in *Atp6v1b2*^R506X/R506X^ mice (^∗^*P* < 0.05 vs. untreated *Atp6v1b2*^R506X/R506X^), reaching levels comparable to WT mice (*P* > 0.05). Data: mean ± SEM (*n* = 6–8 mice/group), one-way ANOVA. **D** Examples of occupancy plots for nonspatial and spatial navigation strategies. Warm colors denote longer occupancy times, whereas cold colors indicate shorter occupancy times. **E** Schematic diagram of the passive avoidance apparatus consisting of light and dark chambers, with a 0.5 mA, 2 s foot shock delivered in the dark chamber during training. Quantification of re-entry latency to the dark chamber (**F**) and retention index (test latency-training latency / test+training latency ratio) (**H**) across wild-type (WT), untreated *Atp6v1b2*^R506X/R506X^, and CPT-cAMP treated *Atp6v1b2*^R506X/R506X^ mice. Both WT and CPT-cAMP treated *Atp6v1b2*^R506X/R506X^ mice had a longer latency time on day 2 compared to day 1 (^∗∗∗^*P* < 0.001; ^∗^*P* < 0.05, Student’s *t*-test) with lower delay index while *Atp6v1b2*^R506X/R506X^ mice showed no significant difference between the two days (*P* > 0.05, Student’s *t*-test). **G** Average escape latency per day during acquisition of Barnes maze (^∗∗^*P* < 0.01; one-way ANOVA; *n* = 8–10 mice/group). Data are presented as mean ± SEM. **I** Mean speed in which spatial or non-spatial strategies and **J** average error counts across acquisition days. (^∗∗∗^*P* < 0.0001; ^∗∗^*P* < 0.01; ^∗^*P* < 0.05, one-way ANOVA). Data are presented as mean ± SEM.

The *Atp6v1b2* c.1516C>T mouse model has previously demonstrated hippocampal-dependent learning and memory deficits through NOR and passive avoidance tests [11]. Recent evidence further indicates that this genetic defect causes vestibular dysfunction [12]. Given the critical role of both hippocampal and vestibular systems in spatial cognition [20], here we conducted a systematic behavioral assessment of *Atp6v1b2*^R506X/R506X^ and CPT-cAMP treatment groups. This behavioral battery – specifically designed to dissect distinct cognitive domains affected by dual hippocampal-vestibular pathology—included NOR, Passive avoidance, and Barnes maze paradigms. Notably, CPT-cAMP administration rescued hippocampal-dependent memory functions in mutant mice. In the NOR paradigm, treated homozygotes demonstrated restored novelty preference, exhibiting a significantly elevated recognition index compared to untreated counterparts (Fig. 7A–C), indicative of recovered object discrimination capacity. The passive avoidance test revealed a critical therapeutic effect on associative learning: While untreated mutants failed to establish context-shock associations (as evidenced by minimal latency changes in entering the shock-paired dark chamber), CPT-cAMP-treated animals showed prolonged entry latencies comparable to wild-type controls (Fig. 7E, F, H), demonstrating preserved aversive memory formation. Spatial navigation deficits, a convergence point of hippocampal and vestibular dysfunction, were comprehensively assessed through Barnes maze performance. Untreated mutants exhibited hallmark spatial learning impairments including protracted escape latencies (Day 5: WT vs HO, ^**^P < 0.01) and elevated error rates. CPT-cAMP treatment effectively normalized these deficits, with trajectory analyses and heatmaps revealing wild-type-like search strategies in treated homozygotes (Fig. 7D). Quantitative metrics confirmed progressive improvement across training days, culminating in escape latencies (Fig. 7G) and error counts (Fig. 7J) indistinguishable from wild-type performance by test completion. Critically, intergroup velocity measurements remained statistically equivalent throughout testing (*P* > 0.05), excluding motor confounds in cognitive interpretation.

These collective findings demonstrate that CPT-cAMP administration achieves multimodal cognitive rescue in *Atp6v1b2* c.1516C>T homozygotes, effectively mitigating hippocampal-dependent memory deficits, restoring associative learning capacity, and normalizing spatial navigation impairments arising from combined neurovestibular pathology.

### CPT-cAMP ameliorates autophagic vesicle accumulation through restoration of autophagic flux

The functional integrity of V-ATPase is essential for maintaining lysosomal acidification and proteolytic activity in neural cells [21]. Previous studies demonstrate that V-ATPase inhibition disrupts lysosomal pH homeostasis and impairs autophagosome-lysosome fusion, a critical step in autophagic degradation [22]. Building on our prior findings of hippocampal neuronal loss and cognitive impairment in *Atp6v1b2*^R506X/R506X^ mice [11], coupled with our demonstration of autophagic flux blockade in *ATP6V1B2*^R506X/+^ and *ATP6V1B2*^R506X/R506X^ cells, we hypothesized that this V-ATPase mutation might induce autophagic dysfunction through impaired clearance of autophagic vesicles in hippocampal neurons.

**Fig. 8:**
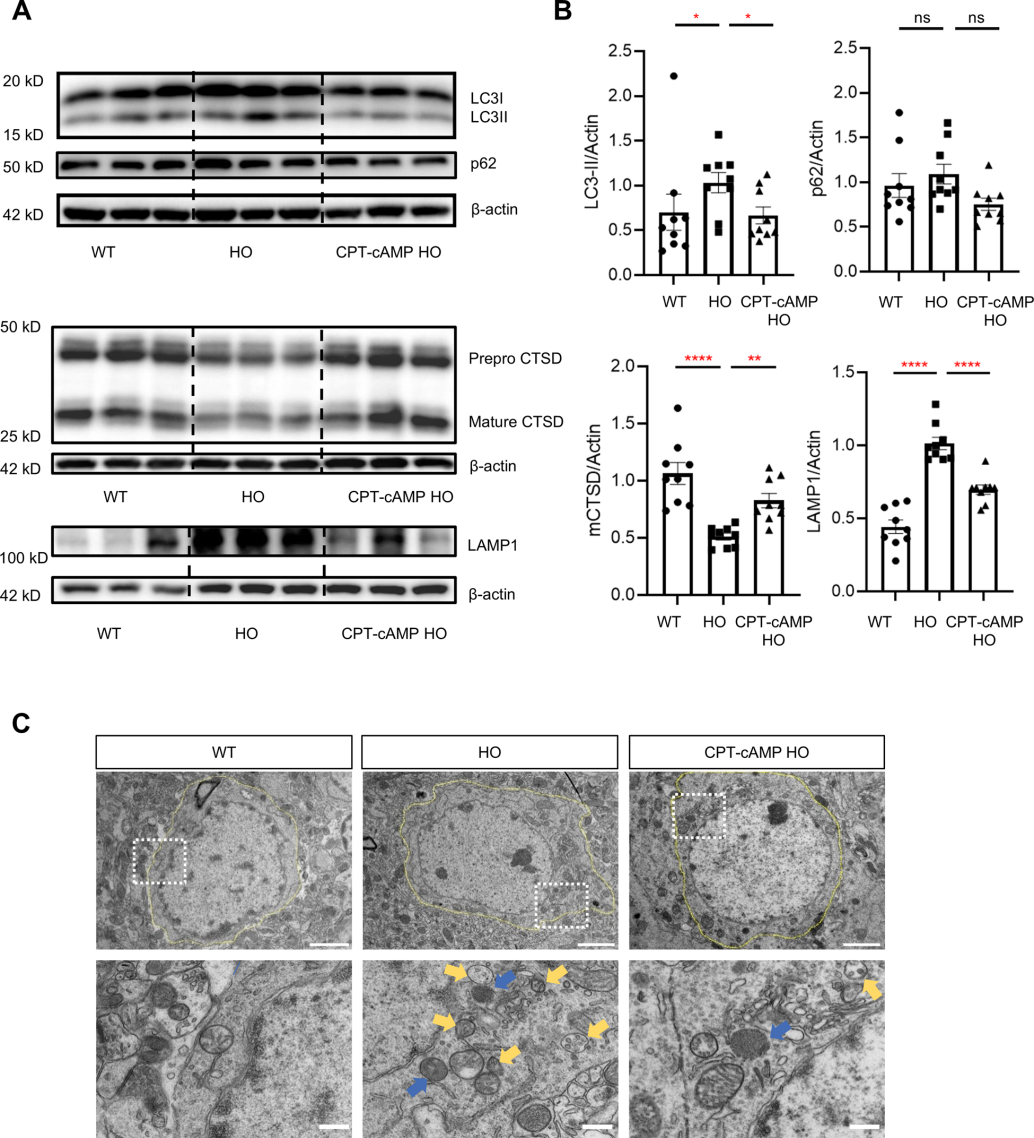
CPT-cAMP rescued accumulation of undegraded autophagosomes in *Atp6v1b2*^R506X/R506X^ mice. **A**, **B** Representative western blot exposure images showing protein expression of LC3I, LC3II, p62, Cathepsin D and LAMP1 in WT, *Atp6v1b2*^R506X/R506X^ mice and CPT-cAMP treated *Atp6v1b2*^R506X/R506X^ mice (*n* = 9 mice/group). **C** Transmission electron microscopy images in hippocampal CA1 region of WT, *Atp6v1b2*^R506X/R506X^ mice and CPT-cAMP treated *Atp6v1b2*^R506X/R506X^ mice at 12 weeks. Neuron of *Atp6v1b2*^R506X/R506X^ mice showed accumulation of autophagosomes (yellow arrows) and lysosomes (blue arrows) in the cytoplasmic region and the treatment group was more relieved in comparison. Scale bars, 2 μm (top) and 500 nm (bottom). Data are presented as the mean ± SEM. ^*^P < 0.05, ^**^P < 0.01, ^***^P < 0.001, ^****^P < 0.0001 by one-way ANOVA, Tukey’s multiple-comparison test.

To investigate autophagic flux dynamics in vivo, we also employed a multimodal approach combining biochemical markers (LC3 isoforms and p62) [23, 24], lysosomal proteolytic capacity (maturation of Cathepsin D), and lysosomal membrane protein (LAMP1 levels) with ultrastructural analysis via transmission electron microscopy (TEM). Western blot analysis revealed a striking accumulation of the autophagosome-associated LC3-II isoform in mutant hippocampal neurons compared to WT controls, where LC3-I remained predominant (Fig. 8A, B). While p62 accumulation did not reach statistical significance, the observed reduction in mature Cathepsin D coupled with marked LAMP1 upregulation in homozygous mice provide complementary evidence supporting autophagic flux impairment and associated lysosomal dysfunction in this model (Fig. 8A, B). This biochemical evidence of autophagosome accumulation was corroborated by TEM observations showing increased numbers of characteristic double-membrane autophagosomes and single-membrane lysosomal structures (Fig. 8C). The concurrent elevation of both autophagic compartments - autophagosomes and lysosomes - suggests a blockade in autophagic maturation rather than enhanced autophagosome formation. This pattern is consistent with impaired autophagosome-lysosome fusion efficiency and subsequent failure of vesicular degradation. Importantly, pharmacological intervention with CPT-cAMP substantially normalized both biochemical and morphological markers of autophagic flux disruption, indicating its therapeutic potential in restoring lysosome-mediated clearance mechanisms.

## Discussion

Our systematic analysis of 36 *ATP6V1B2*-related syndrome cases revealed striking neuroclinical predominance, with epilepsy manifesting in 69.44% (25/36) of patients and cognitive impairment affecting 83.33% (30/36). This distinctive dual-phenotype clustering establishes *ATP6V1B2* deficiency as a unique nosological entity within the spectrum of V-ATPase disorders, which typically demonstrate more heterogeneous neurological presentations. To mechanistically bridge this genotype-phenotype correlation, we developed a knock-in mice model (*Atp6v1b2*^R506X/R506X^) that faithfully recapitulates both spontaneous epileptogenesis and hippocampus-dependent cognitive deficits. The phenotypic convergence in both humans and mice provides compelling evidence for a unified etiological mechanism - lysosomal acidification failure - underlying these seemingly disparate neurological manifestations.

Current therapeutic approaches for lysosomal pH dysregulation remain fundamentally indirect, often targeting downstream consequences rather than the primary defect. For instance, in conditions characterized by insufficient lysosomal acidification, such as non-alcoholic fatty liver disease (NAFLD) [25], mTOR inhibitors like rapamycin have been employed to enhance autophagic flux, thereby upregulating V-ATPase expression and restoring lysosomal acidity [26]. Our prior work demonstrated that systemic administration of apoptosis inhibitors could partially rescue spiral ganglion neuron dysfunction in *Atp6v1b2*^R506X/R506X^ mice [27], suggesting secondary cellular pathways may be pharmacologically tractable. However, these strategies share a critical limitation: none directly address the root cause of impaired proton translocation through the V-ATPase complex itself. This therapeutic gap underscores the urgent need for molecular interventions capable of specifically enhancing V-ATPase-mediated acidification, particularly given the centrality of lysosomal pH in maintaining proteostatic equilibrium and synaptic function or neuronal excitability.

In this study, we established an isogenic knock-in cellular model carrying the pathogenic variant, *ATP6V1B2* p.R506X. Through systematic characterization of this model, we mechanistically elucidated how *ATP6V1B2* deficiency disrupts lysosomal homeostasis. To precisely quantify lysosomal pH dynamics, we employed a ratiometric, genetically-encoded biosensor [17] which directly demonstrated *ATP6V1B2*’s indispensable role in V-ATPase-mediated acidification. This finding aligns with previous studies that implicated lysosomal dysfunction in neurodegenerative diseases, reinforcing the relevance of our model for studying disease mechanisms and potential therapeutic interventions. Combining our previous research findings [10, 11, 27], we have confirmed that the *ATP6V1B2* p.R506X disrupts lysosomal acidification by impairing V-ATPase proton pump functionality, likely through destabilizing subunit assembly or compromising proton channel architecture.

Our initial characterization revealed an inverse correlation between truncated protein length and the severity of lysosomal acidification defects across three engineered cell lines. Based on the pathophysiological relevance of *ATP6V1B2*^R506X/+^ cells - which recapitulate the human mutation spectrum observed in *ATP6V1B2*-associated syndromic disorders - we focused subsequent pharmacological screening on this model system. A multi-tiered therapeutic strategy was implemented to address distinct aspects of mutation-induced lysosomal pathophysiology. Membrane-permeable cAMP analog has been verified to enhance V-ATPase assembly and proton channel functionality through PKA-mediated phosphorylation of pump subunits [28]. Therefore, we hypothesize that CPT-cAMP may similarly enhance V-ATPase assembly and proton channel function through this conserved signaling mechanism. Rapamycin, an mTOR-dependent autophagy inducer, addresses secondary lysosomal storage pathology by enhancing substrate clearance in alkaline hydrolase-deficient lysosomes [29]. Teprenone, the geranylgeranylacetone derivative, provides microenvironmental stabilization through lysosomal membrane reinforcement, counteracting alkalinization-induced osmotic vulnerability [30]. Strikingly, CPT-cAMP demonstrated superior therapeutic efficacy in both heterozygous (*ATP6V1B2*^R506X/+^) and homozygous (*ATP6V1B2*^R506X/R506X^) mutant lines, achieving statistically significant lysosomal pH normalization. While Rapamycin and Teprenone showed marginal pH improvement trends in heterozygous cells, their effects failed to reach statistical significance compared to untreated controls. The marked superiority of CPT-cAMP underscores the therapeutic primacy of etiological correction over compensatory approaches, likely stemming from its direct mechanistic synergy with the V-ATPase assembly process disrupted by p.R506X mutations. Furthermore, the CPT-cAMP’s penetration of the BBB was confirmed in mice.

Next, we investigated the therapeutic potential of CPT-cAMP in mitigating seizure susceptibility in *Atp6v1b2*^R506X/R506X^ mice. To systematically evaluate anti-epileptogenic efficacy, we employed a pentylenetetrazole (PTZ)-induced seizure paradigm and quantified behavioral manifestations using a modified Racine scale. Our in vivo studies revealed that CPT-cAMP administration substantially attenuated the exacerbated seizure susceptibility in this genetic model, providing mechanistic evidence linking lysosomal acidification deficits to neuronal hyperexcitability. The observed phenotypic rescue (evidenced by reduced seizure scores) suggests that pH homeostasis restoration may disrupt the pathogenic cascade connecting lysosomal dysfunction to network-level excitability abnormalities. Notably, this therapeutic effect parallels clinical observations in human V-ATPase deficiencies [21], where neurological symptoms frequently constitute primary disease manifestations, thereby reinforcing the translational potential of our approach.

Western blot analysis demonstrated a marked decrease in LC3-II levels following CPT-cAMP treatment, indicative of restored autophagic flux. This observation supports two non-exclusive mechanistic interpretations: (1) Improved lysosomal acidification may reactivate pH-dependent hydrolases (e.g., cathepsins), facilitating autophagosome content degradation; and/or (2) V-ATPase functional recovery might alleviate lysosomal stress signals that trigger compensatory autophagy initiation. Corroborating these findings, hippocampal neurons exhibited reduced autophagosome accumulation in treated animals. Collectively, our data establish that CPT-cAMP-mediated restoration of lysosomal acidification normalizes autophagic processing, ultimately reducing seizure susceptibility in *Atp6v1b2*^R506X/R506X^ mice.

While our study demonstrates promising therapeutic effects of CPT-cAMP in rescuing lysosomal pH, cognitive impairment and seizure susceptibility, several limitations warrant consideration. First, validating these findings in multiple independent cell lines or, ideally, in human iPSC-derived neurons would be essential to rule out cell-line-specific effects and significantly strengthen the robustness and translational relevance of our conclusions. Secondly, we hypothesize that CPT-cAMP may affect cellular functions through PKA-independent pathways (such as the Epac pathway), and its regulation of other V-ATPase subunits (such as *ATP6V1A*) cannot be completely ruled out. The pleiotropic effects of CPT-cAMP necessitate further mechanistic dissection to exclude off-target contributions. Thirdly, although we assessed the impact of the drug on body weight up to 12 weeks, the evaluation of chronic toxicity risks, especially lysosomal over-acidification, deserves attention. Finally, there remains a lack of direct in vivo pH measurement in the mouse brain, a central goal for future research that will likely depend on novel pH indicators for in vivo imaging.

In conclusion, this study provides the first preclinical evidence that pharmacological enhancement of V-ATPase assembly via cAMP signaling effectively reverses lysosomal alkalinization and its neurological sequelae. The multi-dimensional rescue spanning molecular defects (pH restoration), cellular pathology (autophagic clearance), and organismal phenotypes (seizure attenuation and cognitive impairment) establishes lysosomal proton pump augmentation as a promising therapeutic axis for *ATP6V1B2*-related encephalopathies. Our findings advocate for a paradigm shift from symptomatic management to etiological intervention in V-ATPase-associated neuropathologies.

## Material and methods

### Study approval

This study received ethical approval from the Institutional Review Board of the Chinese PLA General Hospital (Protocol S2016–120–02). Written informed consent was obtained from all patients or their legal guardians. All animal experiments adhered to the Chinese Association for Laboratory Animal Science (CALAS) guidelines and were approved by the Institutional Animal Care and Use Committees of the Chinese PLA General Hospital and Chinese Institute for Brain Research.

### Case identification and data acquisition

We performed a retrospective cohort study to establish genotype-phenotype correlations in *ATP6V1B2*-related syndromes. The cohort included newly enrolled cases from the genetic disorders registry at the Chinese PLA General Hospital and published cases retrieved from PubMed, OMIM, and ClinVar databases.

### In vitro experiments

#### Design of sgRNA and homology-directed repair template

To generate a nonsense mutation (c.1516C>T) in exon 14 of the *ATP6V1B2* gene (GenBank: NM_001693.4), a single guide RNA (sgRNA) targeting the locus was designed using the CRISPOR Design Tool (http://crispor.tefor.net) with stringent off-target filtering (score >90) [31]. The 20-nucleotide protospacer sequence (5′-aatgctttgcagagtctcga-3′) was synthesized and cloned into the pX458 vector (pSpCas9(BB)-2A-GFP; Addgene #48138) via BbsI restriction enzyme-mediated single-step ligation. A 144-nucleotide single-stranded donor oligonucleotide (ssODN; Tsingke Biotechnology) was designed with homology arms (Left arm:81-bp; Right arm: 62-bp) flanking the mutation site to facilitate precise homology-directed repair (HDR). The ssODN sequence (5′-attggctggcagctactccgaatcttccccaaagaaatgctgaagagaatccctcagagcaccctcagcgaattttaccctTgagactctgcaaagcattagctgctgcttctgcattgctccgcgctcttgtgaaatactggt-3′) incorporated the c.1516T allele (uppercase) and was validated by Sanger sequencing prior to use.

#### Cell culture and genome editing

HEK293T cells (provided by Vector Core, Chinese Institutefor Brain Research, Beijing) were maintained in DMEM (Gibco) supplemented with 10% FBS (Gibco) and 1% penicillin-streptomycin (Gibco) at 37 °C under 5% CO₂. For CRISPR/Cas9 editing, cells at 70–80% confluence were co-transfected with 1 μg pX458-sgRNA plasmid and 10 μM ssODN using jetPRIME® transfection reagent (Polyplus), following manufacturer protocols. Six hours post-transfection, media was replaced to minimize reagent cytotoxicity. Enhanced GFP-positive cells were isolated 48 h later via single-cell sorting (FACSAria III; BD Biosciences) into 96-well plates. Clonal expansion proceeded for 10–14 days prior to genotyping. All experiments were performed with mycoplasma-free cells, as confirmed by regular PCR-based testing.

#### Genotypic validation

Genomic DNA from 56 clonal lines was PCR-amplified using primers flanking the target site (forward: 5′-ggtccttacgaaaatcgcac-3′; reverse: 5′-gagggagttgagaggtaaggtc-3′). Amplicons were sequenced (Tsingke Biotechnology) to confirm the c.1516C>T substitution. Positive clones were expanded for functional assays.

#### Ratiometric lysosomal pH measurement

pCAG RpH-LAMP1-3×FLAG was a product from Massimiliano Stagi (Addgene plasmid # 163018; http://n2t.net/addgene:163018; RRID:Addgene_163018). Cells transiently expressing the pH-sensitive reporter RpH-LAMP1-3×FLAG (Addgene #163018) were plated on glass-bottom dishes and equilibrated for 10 min at 37°C in calibration buffers (pH 4.0–7.0) containing 10 μM nigericin and monensin (Sigma-Aldrich). Live-cell imaging was performed on a Zeiss LSM 880 confocal microscope (63×/1.40 NA oil objective) using dual excitation (488 nm/561 nm) and emission filters (EGFP: 500–550 nm; mRFP: 570–620 nm). Fluorescence ratios (488/561 nm) were quantified in Fiji/ImageJ (v2.3.0) and normalized to pH calibration curves. All lysosomal pH measurements were performed exclusively in live cells.

#### Cytotoxicity assessment

Cell viability was evaluated via MTT assay. Briefly, 8 × 10⁴ cells/well (96-well plate) were treated with test compounds for 6 h, incubated with 10 μL 5 mg/mL MTT (Sigma-Aldrich) for 4 h, and lysed with 100 μL DMSO. Absorbance at 570 nm was measured (BioTek Synergy H1). Viability was expressed as percentage of untreated controls (set to 100%).

### In vivo experiments

Our study examined male and female animals, and similar findings are reported for both sexes. *Atp6v1b2*^R506X/R506X^ mice were randomly divided into CPT-cAMP group and untreated group, and the WT mice were served as baseline controls.

### Generation of *Atp6v1b2*^R506X/R506X^ Mice

The *Atp6v1b2*^R506X/R506X^ mouse line was generated on a C57BL/6 genetic background through CRISPR/Cas9-mediated genome editing by Shanghai Model Organisms Center, Inc. (Shanghai, China), as previously detailed [11]. Homozygous mutants (HO) were obtained by crossing heterozygous (HE) breeding pairs, with littermates serving as wild-type (WT) controls.

### Liquid chromatography-tandem mass spectrometry (LC-MS/MS)

Adult C57BL/6 mice received intraperitoneal injections of CPT-cAMP (20 mg/kg). At 2 h post dosing, plasma and whole brains were collected (*n* = 5). At 2 h post injection (the time point determined from preliminary pharmacokinetic profiling), animals were anesthetized with isoflurane. Blood samples (approximately 800 μL) were collected via cardiac puncture into EDTA-coated tubes and centrifuged at 3000 × *g* for 15 min at 4 °C to obtain plasma. Immediately thereafter, whole brains were excised, rapidly rinsed in ice-cold phosphate-buffered saline (PBS), weighed, and snap-frozen in liquid nitrogen. Drug concentrations in plasma and brain homogenates were quantified by liquid chromatography-tandem mass spectrometry (LC-MS/MS) using CPT-cAMP as an internal standard. Calibration curves (10–1000 μg/mL) demonstrated method validity (accuracy: 98.9–101.6%). The brain-to-plasma ratio (Kp) was calculated as: Brain concentration (μg/g)/Plasma concentration (μg/mL).

### Surgical implantation of EEG electrodes

Mice underwent aseptic stereotaxic surgery under inhalation anesthesia. Animals were initially induced with 3% isoflurane (RWD Life Science Co., Shenzhen, China; cat. #R510-22-10) in 100% oxygen (flow rate: 1 L/min) using a precision vaporizer, followed by maintenance at 1.5% isoflurane via a nose cone integrated with a stereotaxic apparatus (Kopf Instruments, Tujunga, CA, USA; Model 940). Core body temperature was continuously monitored and maintained at 37.0 ± 0.5 °C using a closed-loop thermal regulation system (Harvard Apparatus, Holliston, MA, USA; #50-7220). Ocular protection was achieved by bilateral application of petrolatum-based ophthalmic ointment (Puralube Vet Ointment, Dechra Veterinary Products). Following cranial depilation with thioglycolate depilatory cream (Nair™, Church & Dwight Co.) and triple-cycle aseptic preparation with alternating 70% ethanol (v/v) and 10% povidone-iodine (Betadine™, Avrio Health LP), a midline sagittal scalp incision (~10 mm) was created using sterile surgical instruments (#10 scalpel blade, Feather Safety Razor Co.). The skull surface was meticulously cleared of periosteal tissue using micro curettes (Fine Science Tools, Foster City, CA, USA; #10074-13). Three bilateral burr holes (1.0 mm diameter) were created using a pneumatic micro-drill system (Reward Medical Instruments, Shenzhen, China; #87001) equipped with a 0.5 mm diamond-coated spherical burr (Hager & Meisinger GmbH, Neuss, Germany; #806314K), maintaining intact dural integrity under microscopic visualization (40× magnification). Custom-fabricated Teflon-coated silver wire electrodes (diameter: 200 μm; impedance: <5 kΩ at 1 kHz) were stereotaxically implanted through the cranial openings and secured with light-cured dental cement (3M ESPE, RelyX Unicem). For neuromuscular recording, two bipolar EMG leads were tunneled subcutaneously to the nuchal musculature. Following the surgical intervention, the mice were returned to their home cage and allowed to recover for 7 days.

### Video-electroencephalography (EEG) recordings and analysis

Seizure severity was quantitatively assessed using a modified Racine staging system (stages 0–4) coupled with synchronized electroencephalographic monitoring. Continuous EEG signals were acquired through a dedicated bioamplifier system with bandpass filtering (0.1–300 Hz), signal amplification (1000× gain), and digitization at 1000 Hz sampling frequency. Electrographic seizures were operationally defined as discrete epochs (minimum 10 s duration) characterized by progressive evolution of rhythmic spike discharges in both frequency (typically 5–15 Hz) and amplitude (≥2.5× baseline), culminating in repetitive burst suppression patterns followed by postictal voltage attenuation.

Interictal epileptiform activity was identified as transient events meeting two essential criteria: (1) sharp-wave morphology with rise time <50 ms and total duration <200 ms, and (2) amplitude ≥2.5 times baseline (calculated from 30 s pre-event period). Spike clusters were defined as ≥3 consecutive sharp waves with inter-spike interval <500 ms. Throughout continuous monitoring (24/7 video-EEG), specific pathogen-free mice were maintained in environmentally controlled chambers (22 ± 1 °C, 55% humidity, 12/12 light cycle) with ad libitum access to gamma-irradiated feed (LabDiet 5053) and autoclaved reverse-osmosis water.

### Assessment of pentylenetetrazol-induced seizure susceptibility

To evaluate epileptogenic propensity, *Atp6v1b2*^R506X/R506X^ homozygous (HO) mice and wild-type (WT) littermates (4–16 weeks old, sex-balanced) received intraperitoneal injections of pentylenetetrazol (PTZ; Sigma-Aldrich #P6500) administered at subconvulsive doses until generalized tonic-clonic seizure (GTCS) onset. Two blinded investigators quantified seizure progression through: (1) latency to first partial clonus (PC), generalized clonus (GC), and tonic convulsion (TC); and (2) maximum seizure severity using a modified Racine scale: Stage 1: Behavioral arrest with abdominal contact to cage floor； Stage 2: Focal clonus (facial/cephalic or forelimb involvement)； Stage 3: Generalized clonus (all limbs + tail) with rearing/falling； Stage 4: Generalized tonic-clonic seizure (GTCS). The susceptibility scores of mice = (0.2) (1/PC latency) + (0.3) (1/GC latency) + (0.5) (1/TC latency) [32]. The higher the epilepsy susceptibility score, the more prone the mice were to epilepsy.

### CPT-cAMP intervention protocol

Chronic treatment with CPT-cAMP (Merck, #116812) was initiated at postnatal week 4. The compound was dissolved in sterile saline (0.9% NaCl) and administered intraperitoneally (20 mg/kg) to *Atp6v1b2*^R506X/R506X^ mice (*n* = 6) weekly until week 12 (total 9 doses). Control groups received vehicle injections following identical protocols. The investigator responsible for administering the drug injections and performing all subsequent behavioral tests was blinded to the group assignments.

### Behavioral assessments

#### Novel object recognition (NOR) test

The NOR test was performed to evaluate hippocampal-dependent recognition memory in mice using a three-phase protocol [33].

*Habituation phase*: Mice were permitted to freely explore a sanitized open-field arena (40 × 40 × 40 cm) for 10 min/day over two consecutive days to acclimate to the testing environment.

*Training phase*: On the third day, two identical objects were symmetrically positioned in the arena. Mice were allowed to explore the objects for 5 min, after which they were returned to their home cages.

*Testing phase*: Following a retention interval (short-term: 1 h; long-term: 24 h), one familiar object (A) was replaced with a novel object (B). Exploratory behavior was video-recorded for 5 min, with exploration defined as snout contact ≤2 cm from the object. Mouse trajectories were annotated via SAM (Segment Anything Model)-based tracking throughout the NOR test. Recognition memory was quantified using the Recognition Index (RI): *T*_novel_ /(*T*_novel_ + *T*_familiar_). *T*_novel_: Number of explorations to novel object during the test phase. *T*_familiar_: Number of explorations to the familiar (previously encountered) object during the testing phase.

#### Passive avoidance (PA) test

Associative fear memory was assessed using an automated two-compartment shuttle box (light/dark chambers, Med Associates Inc.).

*Acquisition trial*: Mice were placed in the illuminated compartment, and the guillotine door was raised after 10 s. Upon entry into the dark compartment, a mild foot shock (0.5 mA, 2 s duration) was delivered through the grid floor.

*Retention trial*: Twenty-four hours post-training, mice were reintroduced to the light compartment, and latency to cross into the dark compartment was recorded (cutoff: 300 s). Retention index was calculated as (*T*_test_−*T*_train_)/(*T*_test_+*T*_train_). Prolonged latency periods were interpreted as enhanced retention of aversive memory.

#### Barnes maze (BM) test

Spatial learning and memory were evaluated using a modified Barnes maze protocol. The apparatus consisted of a circular PVC platform (92 cm diameter, 18 equidistant 5-cm holes) elevated 90 cm above the floor, with one target hole connected to a hidden escape box.

##### Habituation

Mice explored the maze for 2 min without the escape box.

##### Acquisition phase (Days 1–4)

Four daily trials (3 min maximum/trial, 15-min inter-trial intervals) were conducted under bright illumination (100 lux). Escape latency (time to locate the target hole) and working memory errors (explorations of non-target holes) were recorded.

##### Probe test (Day 5)

The escape box was removed, and mice freely explored the maze for 120 s. Spatial memory retention was analyzed by quantifying time spent in the target quadrant and frequency of target hole investigations.

##### Control measures

Distal visual cues (geometric patterns) were positioned around the maze to facilitate spatial navigation. Locomotor activity was assessed by calculating mean movement speed (cm/s) across all trials.

### Data inclusion and exclusion criteria

The final sample sizes (*n*) for each behavioral test are reported in the figure legends. To ensure the validity of the data, pre-established exclusion criteria were applied prior to data analysis. Data from an animal were excluded only in cases of significant behavioral disturbance (e.g., freezing for >90% of the session time or failure to initiate exploration) or technical failure (e.g., equipment malfunction or sudden loud noise). All statistical analyses were performed on the final dataset, and the reported significant differences remained robust after these exclusions.

### Transmission electron microscopy (TEM)

Mice were deeply anesthetized with Avertin (0.2 ml/10 g) and transcardially perfused with ice-cold 4% paraformaldehyde (PFA) in 0.1 M phosphate buffer (pH 7.4). Brains were rapidly dissected, and hippocampal regions microdissected in chilled PBS. Tissue blocks (1 mm³) underwent sequential fixation: primary fixation in 2.5% glutaraldehyde/4% PFA in 0.1 M cacodylate buffer (pH 7.4) at 4 °C for 24 h, followed by three 10-min PBS washes. Secondary fixation was performed with 1% osmium tetroxide in 0.1 M cacodylate buffer for 1 h at room temperature.

Specimens were dehydrated through an ethanol series (50%, 70%, 90%, 100%; 15 min per step) followed by acetone infiltration. Tissues were embedded in Epon 812 resin (SPI Supplies) via graded resin:acetone mixtures (1:3, 1:1, 3:1) with 24 h polymerization at 60°C. Ultrathin sections (70–90 nm) were cut using a Leica UC7 ultramicrotome with a diamond knife (Diatome), mounted on 200-mesh copper grids, and double-stained with 2% uranyl acetate (15 min) and Reynolds’ lead citrate (5 min). Imaging was performed on a Hitachi H-7650B TEM operated at 80 kV with an AMT XR-41 CCD camera. A minimum of three biological replicates per group were analyzed, with ≥10 fields captured per sample.

### Western blot analysis

Hippocampal tissues were homogenized in RIPA buffer (50 mM Tris-HCl pH 7.4, 150 mM NaCl, 1% NP-40, 0.5% sodium deoxycholate, 0.1% SDS) containing protease/phosphatase inhibitors (Roche). Protein concentrations were determined by BCA assay (Pierce). Equal protein loads (30 μg) were resolved on 12% Tris-glycine gels (Applygen, B1027) and transferred to PVDF membranes (Millipore) using a semi-dry system (Bio-Rad).

Membranes were blocked with 5% non-fat milk in TBST (Tris-buffered saline with 0.1% Tween-20) for 1 h at room temperature, then incubated with primary antibodies against LC3 (1:1000, CY5992, Abways), p62/SQSTM1 (1:1000, CY5546, Abways), Cathepsin D (1:1000, 69854, CST) and LAMP1(1:1000, sc-20011, Santa Cruz Biotechnology) in blocking buffer at 4 °C for 16 h. After three 10-min TBST washes, membranes were incubated with HRP-conjugated secondary antibodies (1:5000, Cell Signaling Technology) for 1 h at room temperature. Chemiluminescent detection was performed using ECL Prime (GE Healthcare) and quantified with ImageLab 6.0 (Bio-Rad). β-actin served as a loading control. Three independent biological replicates with technical duplicates were analyzed.

### Statistical analysis

All statistical analyses were performed using GraphPad Prism software (version 8.0). Quantitative data are presented as mean ± standard error of the mean (SEM). For Western blot quantification, densitometric analysis was conducted using ImageJ software. In imaging experiments, a minimum of 6–8 fields of view per condition were analyzed from three biological replicates.

Between-group comparisons were performed using the Student’s *t*-test (two-tailed) for two-group comparisons, while one-way ANOVA followed by Tukey’s multiple-comparison was applied for multiple comparisons. Statistical thresholds were defined as follows: *P* < 0.05, ^**^P < 0.01, ^***^P < 0.001, and ^****^P < 0.0001.

## Supplementary information

Supplementary Figure 1

Supplementary Figure 2

Supplementary Figure 3

Supplementary Figure 4

Supplementary figure legend

Full unedited blot

## Data Availability

All data supporting the conclusions of this study are included in the article and its supplementary materials.
