# Supplementary figures and images for: Therapeutic potential of cAMP-mediated lysosomal pH modulation in ATP6V1B2-related neuropathology
Source: Cell Death Discov. 2026 Mar 27;12:199. doi: 10.1038/s41420-026-03056-4 (PMC13150000; doi:10.1038/s41420-026-03056-4)

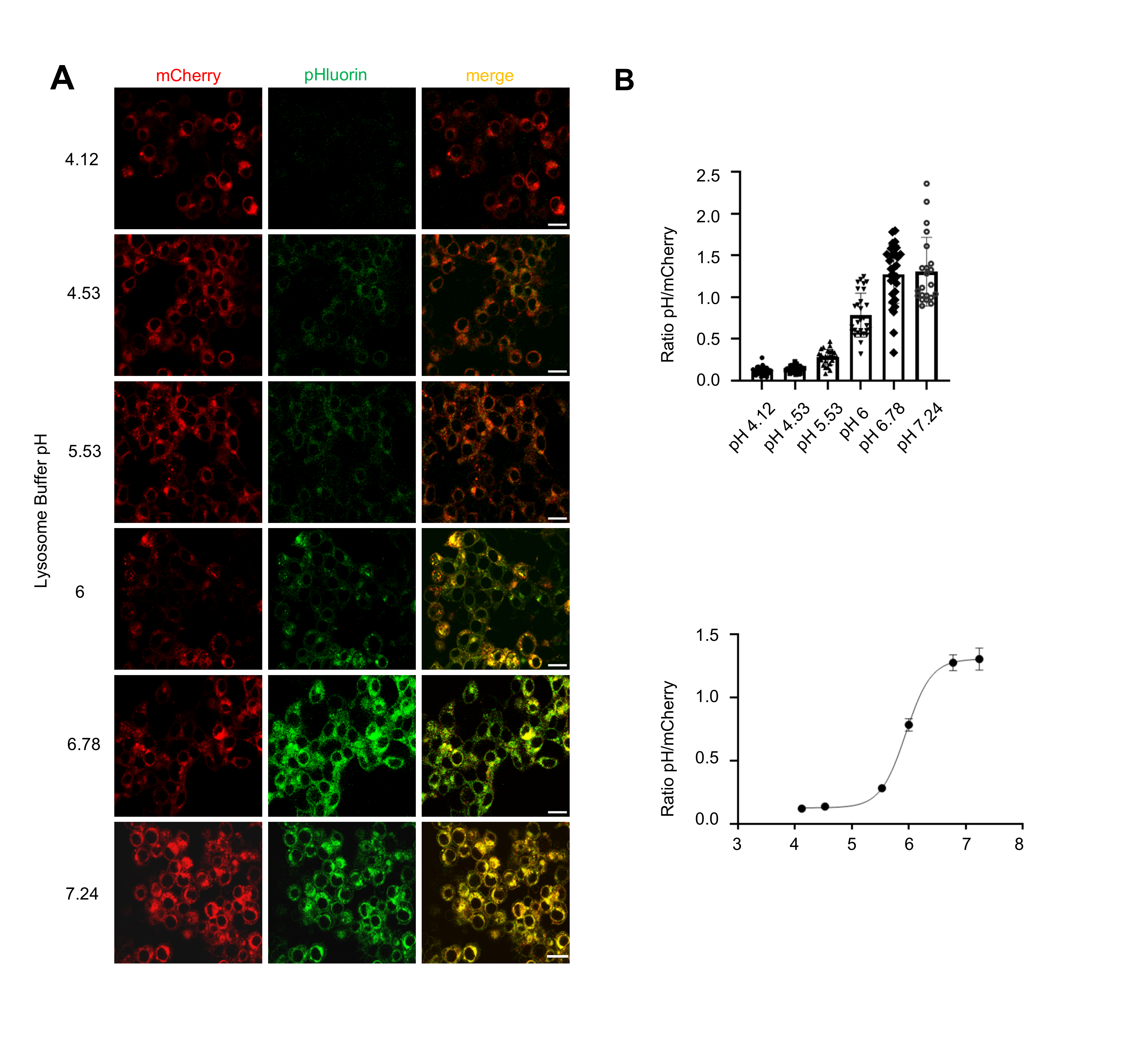

Supplement: Supplementary file 1 — Supplementary Figure 1 [file 41420_2026_3056_MOESM1_ESM.tif]

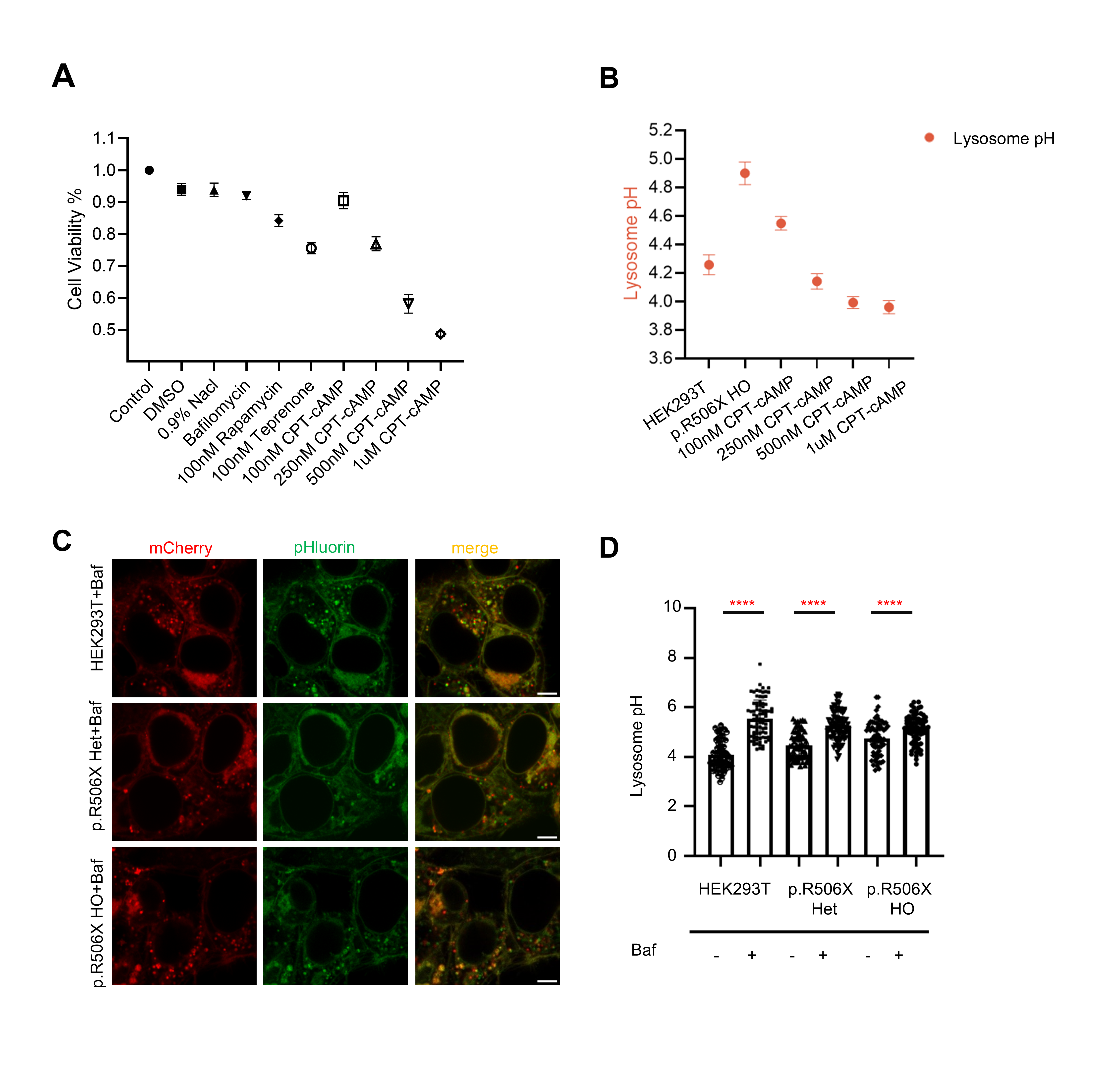

Supplement: Supplementary file 2 — Supplementary Figure 2 [file 41420_2026_3056_MOESM2_ESM.tif]

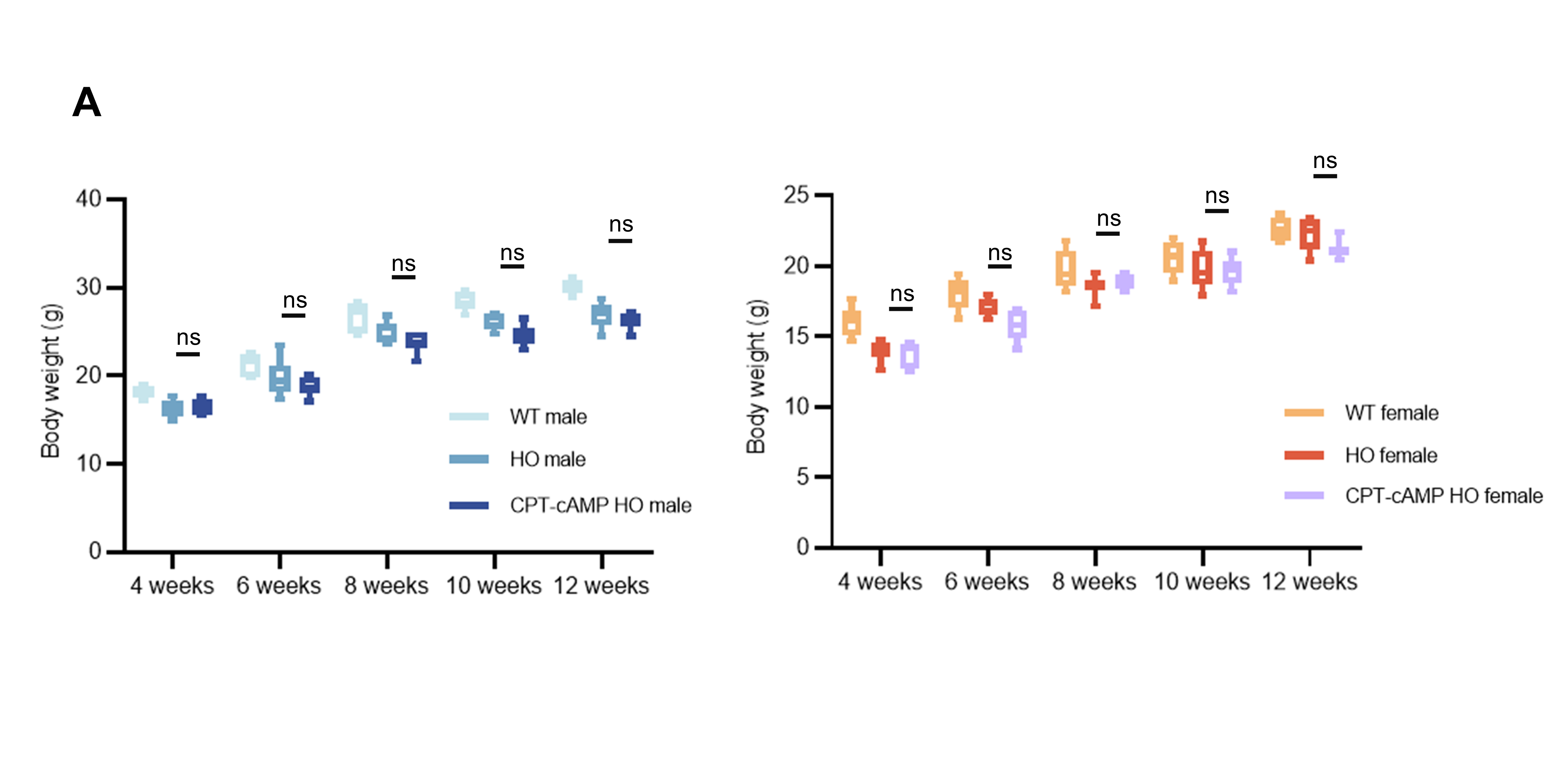

Supplement: Supplementary file 3 — Supplementary Figure 3 [file 41420_2026_3056_MOESM3_ESM.tif]

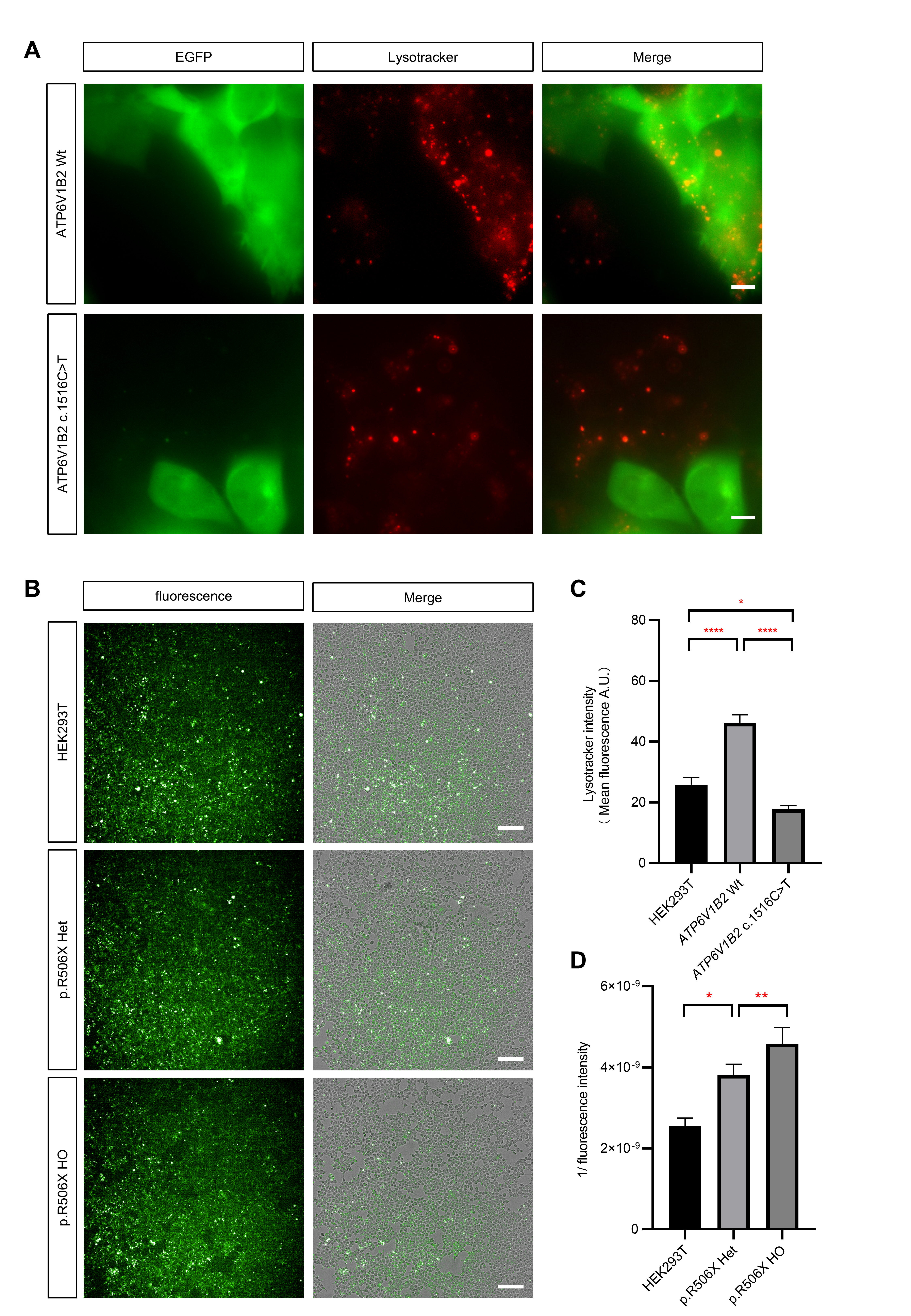

Supplement: Supplementary file 4 — Supplementary Figure 4 [file 41420_2026_3056_MOESM4_ESM.tif]
