# Supplementary material for: Therapeutic potential of cAMP-mediated lysosomal pH modulation in ATP6V1B2-related neuropathology
Source: Cell Death Discov. 2026 Mar 27;12:199. doi: 10.1038/s41420-026-03056-4 (PMC13150000; doi:10.1038/s41420-026-03056-4)
Supplement: Supplementary file 5 — Supplementary figure legend [file 41420_2026_3056_MOESM5_ESM.docx]

**Figure S1. Calibration of RpH-LAMP1-3×FLAG sensor.** (A) Calibration series in live-imaged HEK293T exposed to a series of buffers at fixed pH showing mCherry and pHluorin channels, merged image. Scale bar: 20 µm. (B) Calibration of RpH-LAMP1-3×FLAG in live-imaged HEK293T cells. Top panel: Ratio of pHluorin to mCherry fluorescence in nigericin plus monensin -treated live cells with external buffers set at the indicated pH. Bottom panel: Generated standard curve based on the experimental data.

**Figure S2.** **Cell viability assay and rescue of abnormal lysosome pH in *ATP6V1B2*^R506X/R506X^ cells by CPT-cAMP.** (A) Effects of 100nM Rapamycin, Teprenone and different concentrations of CPT-cAMP on cell viability. (B) Quantification of average lysosomal pH in *ATP6V1B2*^R506X/R506X^ cells treated with different CPT-cAMP concentrations (*n* = 42-73/group). (C) Representative confocal images of HEK293T, *ATP6V1B2*^R506X/+^ and *ATP6V1B2*^R506X/R506X^ cells treated by Bafilomycin A1(Baf) to assess lysosome pH. Scale bar: 5µm. (D) Mean lysosomal pH of HEK293T, *ATP6V1B2*^R506X/+^ and *ATP6V1B2*^R506X/R506X^ cells in the presence or absence of Bafilomycin A1(Baf) (b,d) (Data are expressed as average ± SEM; ∗*P* < 0.05, ∗∗*P* < 0.01, ∗∗∗*P* < 0.001, ∗∗∗∗*P* < 0.0001; one way ANOVA).

**Figure S3.** **CPT-cAMP exhibited a favorable safety profile in vivo.** (A) Body weight of *Atp6v1b2*^R506X/R506X^ mice after injection of CPT-cAMP at different ages. Body weight did not change significantly as compared to control after injection. (*n* = 6 mice/group).

**Figure S4. Mutant ATP6V1B2 impairs lysosomal acidification across different assay.** (A) Lysosomal acidity assessment in HEK293T cells transiently transfected with either wild-type or mutant ATP6V1B2 plasmid. Representative fluorescence images of cells stained with LysoTracker Red are shown. Scale bar: 5 µm. (B) The fluorescence intensity of a commercial lysosomal pH indicator was measured in genetically engineered cell lines to determine lysosomal pH. Representative fluorescence images are shown for HEK293T, *ATP6V1B2*^R506X/+^ cells and *ATP6V1B2*^R506X/R506X^ cells. Scale bar: 100 µm. (C) Quantification of Lysotracker intensity revealed a significantly weakened fluorescence signal in mutant-expressing cells, indicating impaired acidification. (D) Quantification of the normalized fluorescence intensity reveals a progressive decrease in signal from HEK293T to *ATP6V1B2*^R506X/R506X^ cells, corresponding to a progressive elevation in lysosomal pH. (Data are expressed as average ± SEM; ∗*P* < 0.05, ∗∗*P* < 0.01, ∗∗∗*P* < 0.001, ∗∗∗∗*P* < 0.0001; one way ANOVA).
