# Supplementary material for: Therapeutic potential of cAMP-mediated lysosomal pH modulation in ATP6V1B2-related neuropathology
Source: Cell Death Discov. 2026 Mar 27;12:199. doi: 10.1038/s41420-026-03056-4 (PMC13150000; doi:10.1038/s41420-026-03056-4)
Supplement: Supplementary file 6 — Full unedited blot [file 41420_2026_3056_MOESM6_ESM.docx]

**Therapeutic Potential of cAMP-Mediated Lysosomal pH Modulation in *ATP6V1B2*-Related** **Neuropathology**

*Lu Zheng^1,2,3,4,5,6^****^†^****, Weihao Zhao^2,3,4,5,6^****^†^****, Guang Yang^7,8^, Shiwei Qiu^9,10^, Yahong Li^10^, Lin Gao^11^, Gege Wei^10^, Ying Ma^2,3,4,5,6^, Jiangping Xie^10^, Xue Gao^10^, Linyan Chen^10^, Xiaoge Li^2,3,4,5,6^, Rongfeng Lin^2,3,4,5,6^, Wei Xiong^*10^, Yongyi Yuan^*2,3,4,5,6^, Pu Dai^*1,2,3,4,5,6^*

Figure 4A


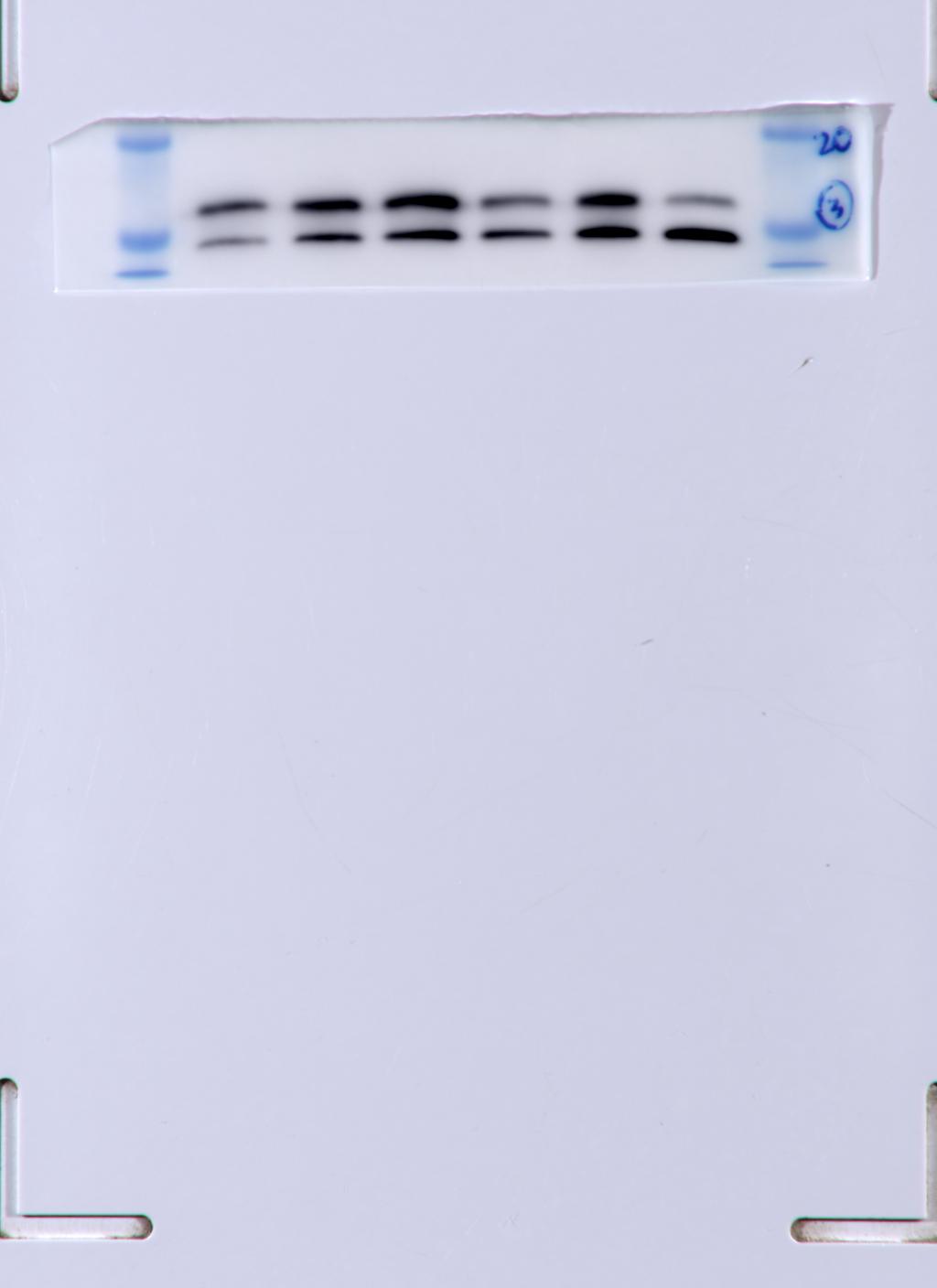


Full unedited blot/gel for Figure 4A LC3I and LC3II.





Full unedited blot/gel for Figure 4A p62.


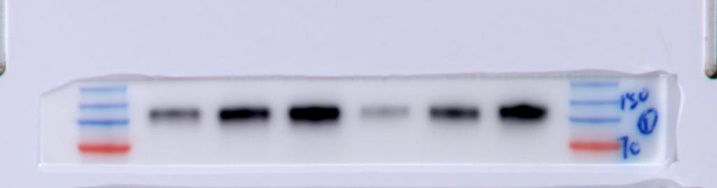


Full unedited blot/gel for Figure 4A LAMP1.


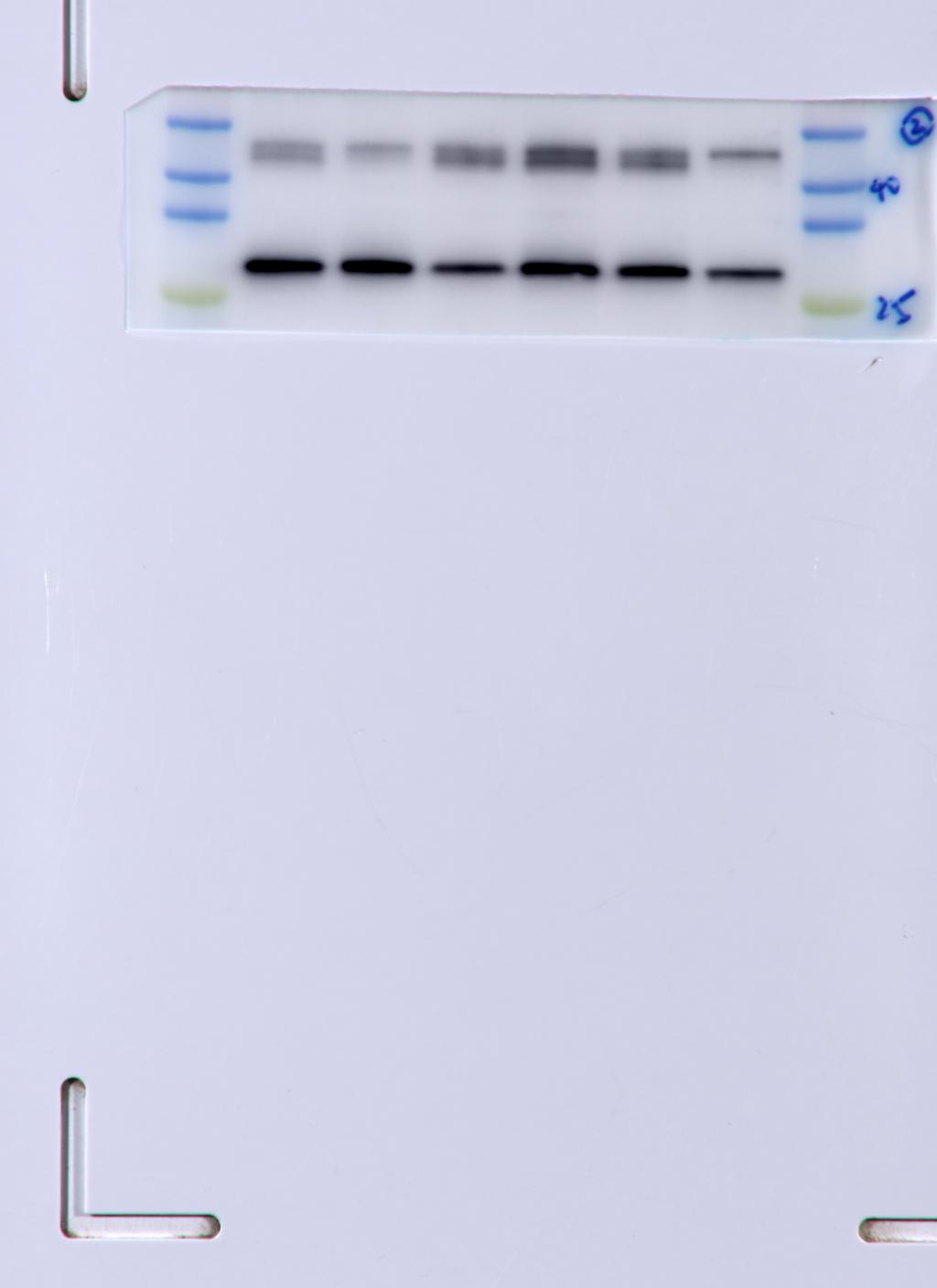


Full unedited blot/gel for Figure 4A CTSD.


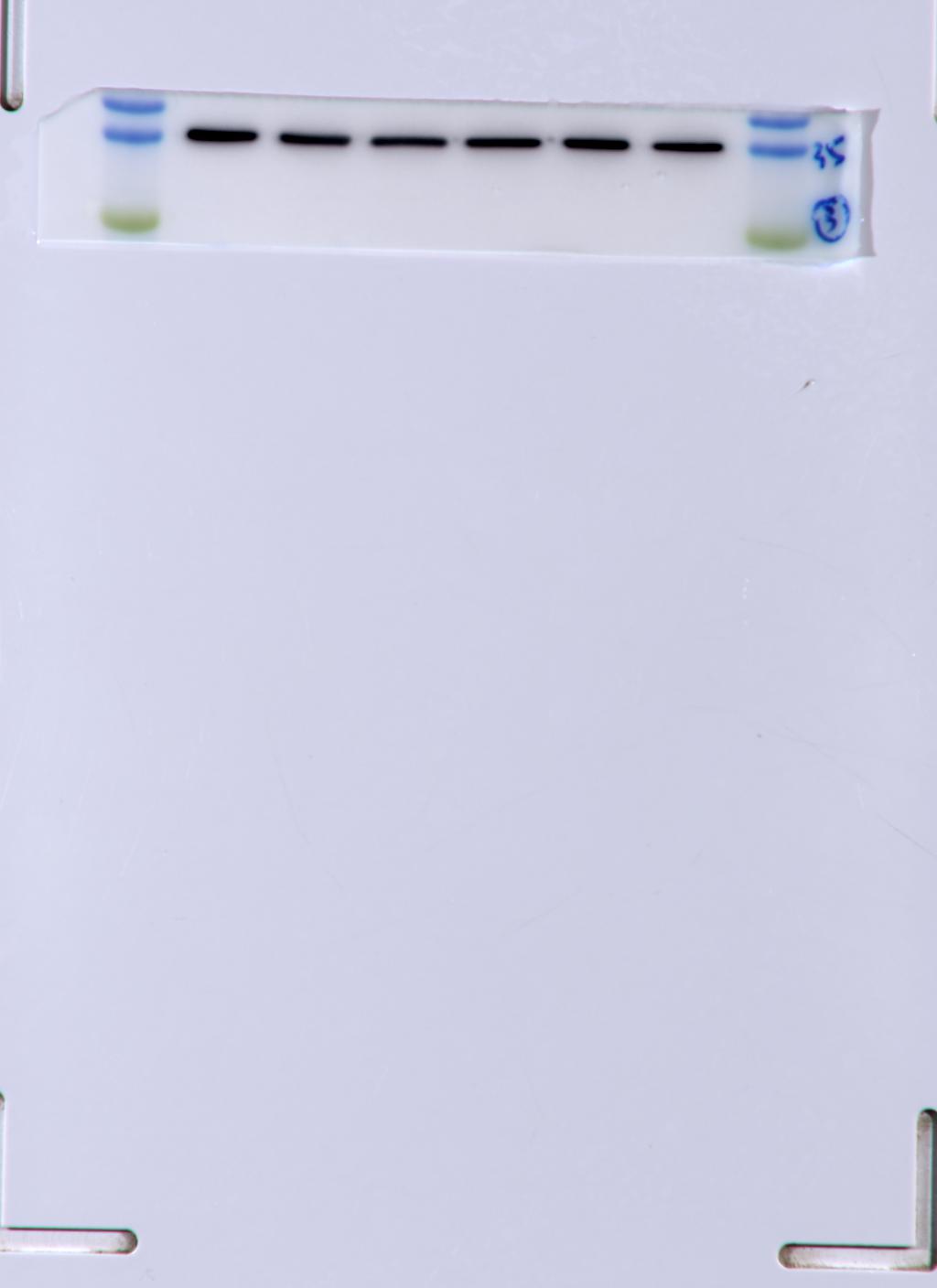


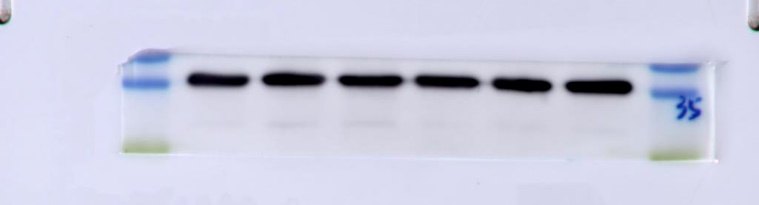


Full unedited blot/gel for Figure 4A GAPDH.

Figure 8A


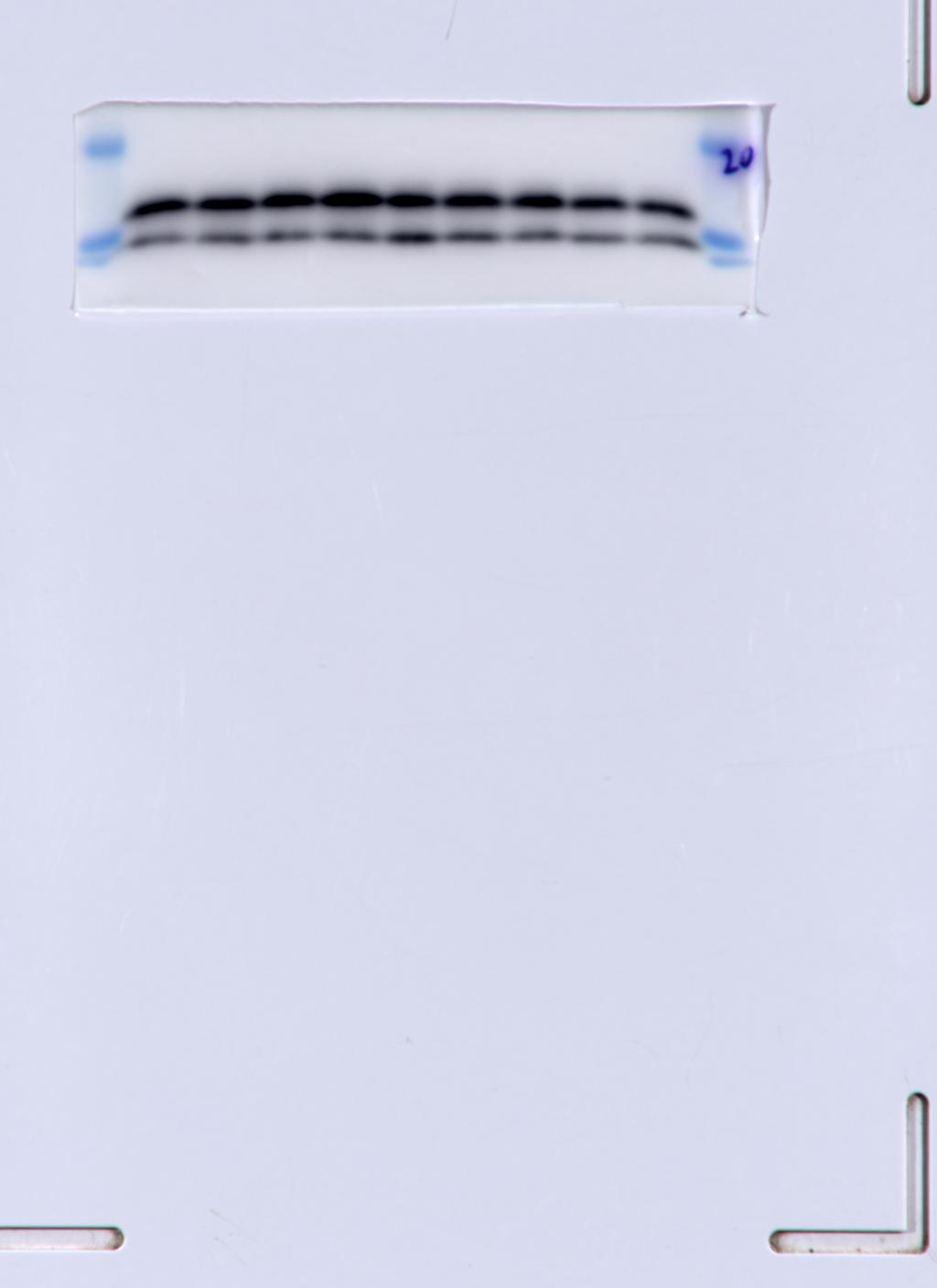


Full unedited blot/gel for Figure 8A LC3I and LC3II.


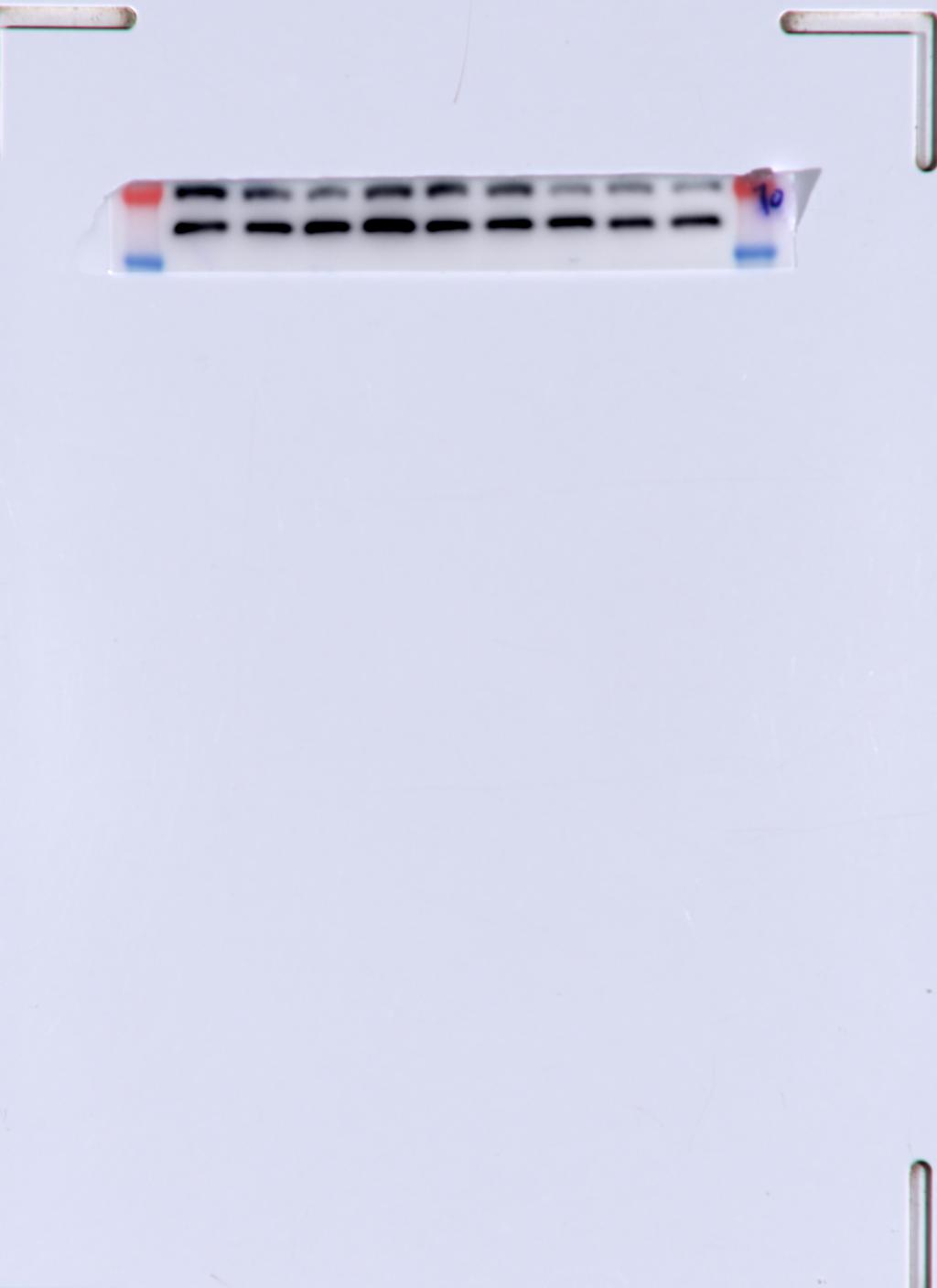


Full unedited blot/gel for Figure 8A p62.


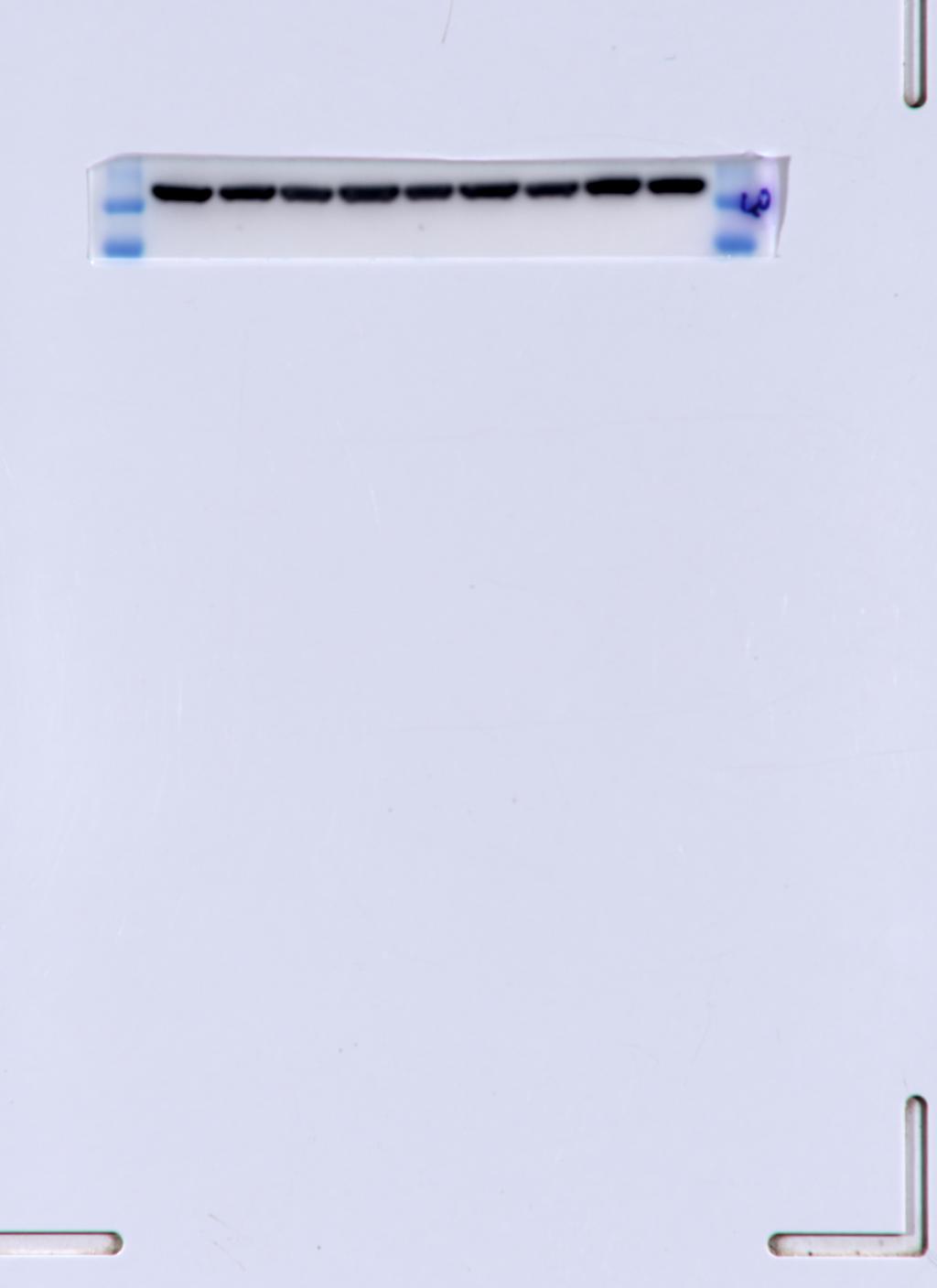


Full unedited blot/gel for Figure 8A actin.


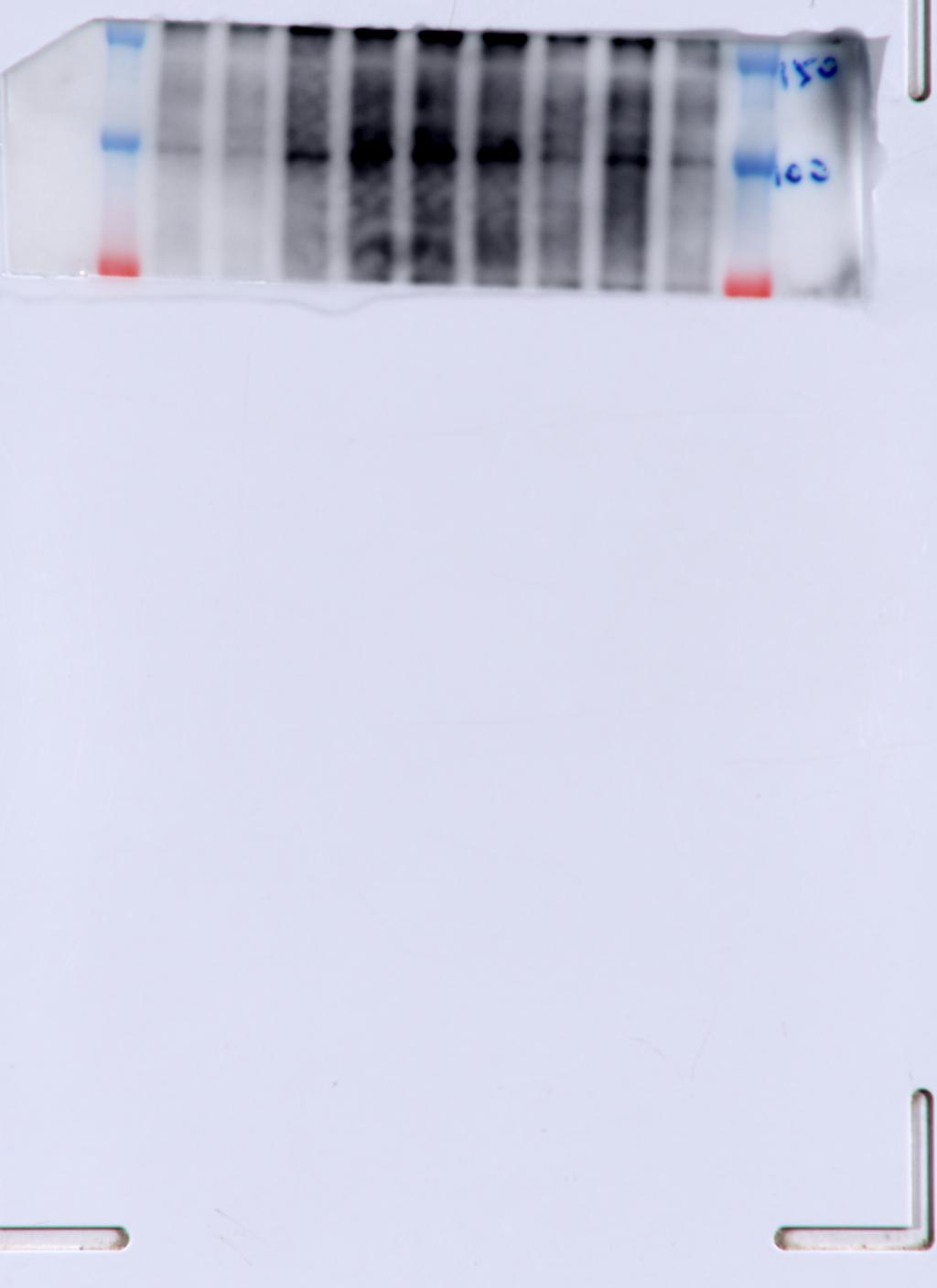


Full unedited blot/gel for Figure 8A LAMP1.


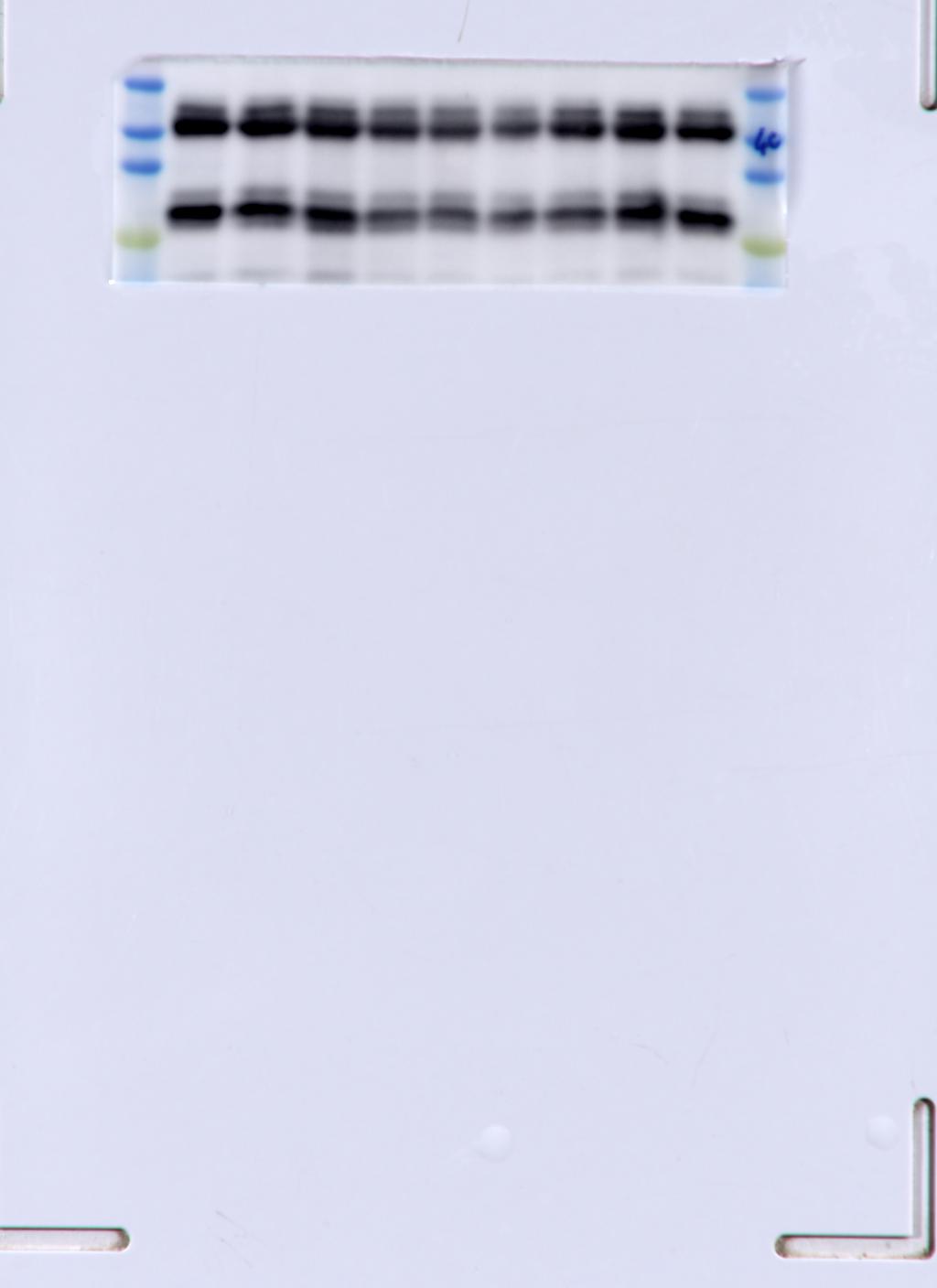


Full unedited blot/gel for Figure 8A CTSD.


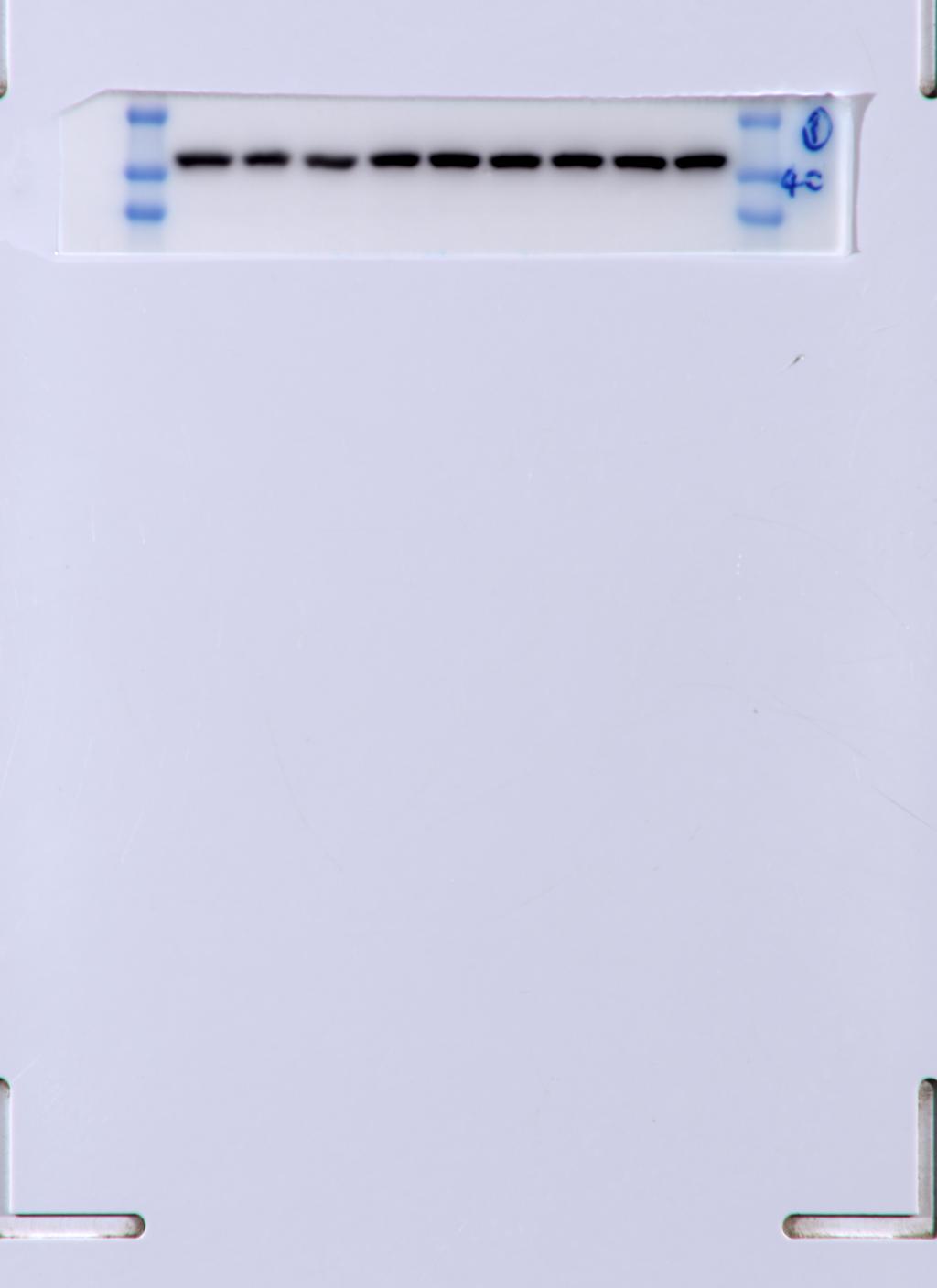


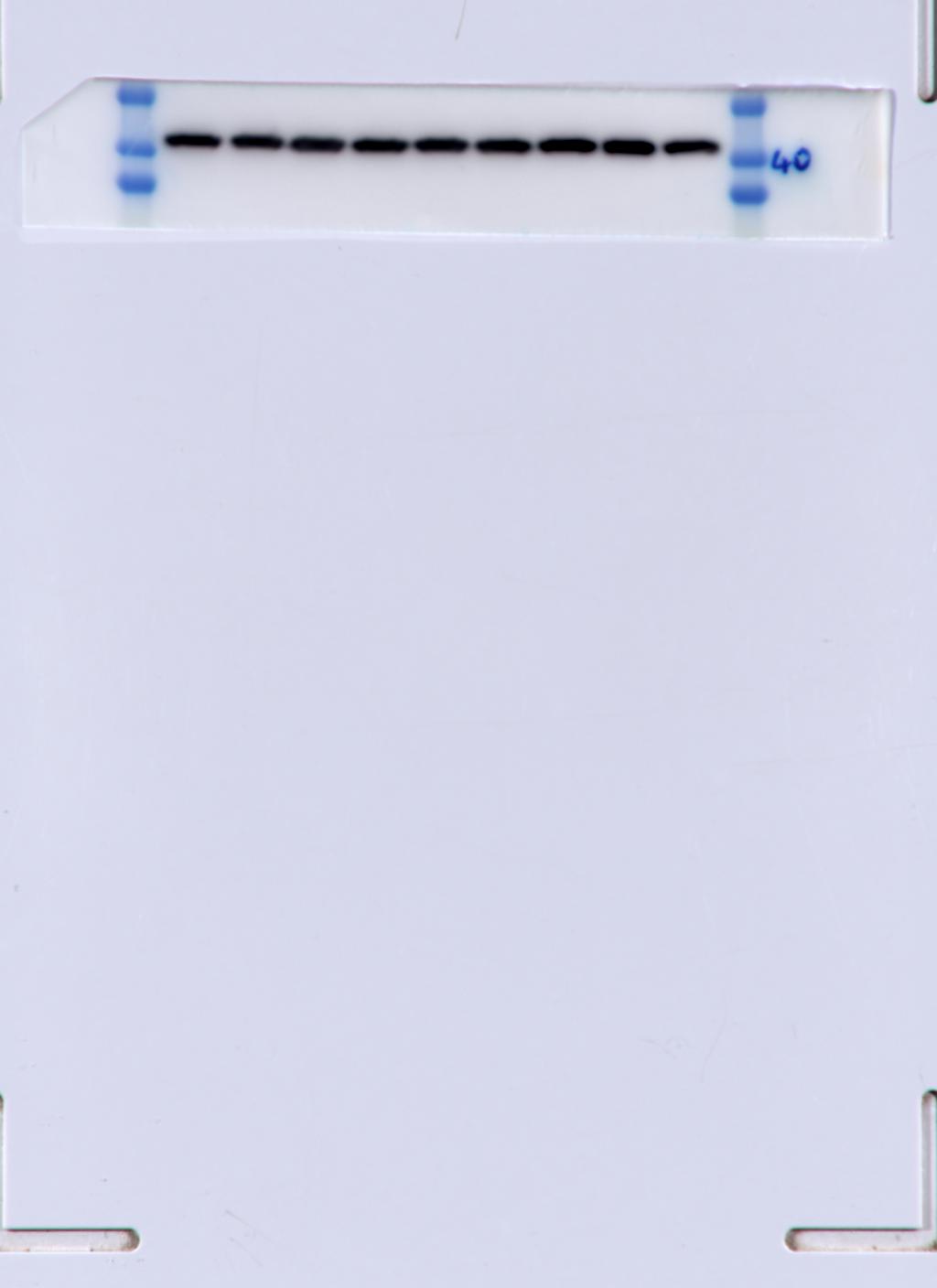


Full unedited blot/gel for Figure 8A actin.
